# Steering from C_1_ to C_2_ Products from the Photocatalytic CO_2_ Conversion over Correlated Single-Atom Pairs

**DOI:** 10.1021/jacs.6c08783

**Published:** 2026-06-17

**Authors:** Biyun Lin, Zixian Li, Zhen Zhan, Ka-Wa Wong, Yunong Li, Shogo Kawaguchi, Shintaro Kobayashi, Tai-Sing Wu, Wei-Min Tu, Yun-Liang Soo, Yufei Zhao, Songhua Cai, Jun Yin, Tsz Woon Benedict Lo

**Affiliations:** † Department of Applied Biology and Chemical Technology, 26680 The Hong Kong Polytechnic University, Hung Hom, Hong Kong 999077, China; ‡ State Key Laboratory of Chemical Resource Engineering, 47832 Beijing University of Chemical Technology, Beijing 100029, China; § Department of Applied Physics, The Hong Kong Polytechnic University, Hung Hom, Hong Kong 999077, China; ∥ 133704 Japan Synchrotron Radiation Research Institute (JASRI), 1-1-1 Kouto, Sayo-cho, Sayo-gun, Hyogo 679-5198, Japan; ⊥ National Synchrotron Radiation Research Center, 101 Hsien-Ann Road, Hsinchu, Taiwan 30076, ROC; # Department of Physics, 34881 National Tsing Hua University, Hsinchu, Taiwan 30013, ROC; ¶ PolyU-Daya Bay Technology and Innovation Research Institute, The Hong Kong Polytechnic University, Huizhou, Guangdong 516081, China; ■ The Hong Kong Polytechnic University Shenzhen Research Institute, The Hong Kong Polytechnic University, Shenzhen 518057, China

## Abstract

The urgent need to mitigate accelerating CO_2_ emissions has driven intense interest in photocatalytic CO_2_ reduction (CO_2_RR), a process that mimics natural photosynthesis to generate carbon-neutral, value-added chemicals. While efficiency has improved, the selective production of high-value C_2+_ products rather than C_1_ compounds remains a critical challenge to economic viability. Single-atom catalysts offer promise for steering selectivity, yet how atomic-scale spatial distribution dictates reaction pathways remains poorly understood. Here, we demonstrate a programmed spatial distribution approach to induce synergistic cooperativity between neighboring Cu motifs within a UiO-67 matrix. By precisely modulating site distribution, we reveal a spatial threshold at which the framework transitions from isolated sites favoring C_1_ products to correlated single-atom pairs (CSAPs) that facilitate C–C bond formation. Temperature-resolved electron paramagnetic resonance spectroscopy provides compelling evidence for the distribution-dependent proximity, capturing a unique magnetic feature that emerges as torsional linker motions are suppressed. This structural configuration utilizes the inherent rotational flexibility of the linkers to dynamically optimize interatomic distances, effectively stabilizing [OC–CO]* dimer intermediates and steering the reaction toward C_2_ products.

## Graphical abstract

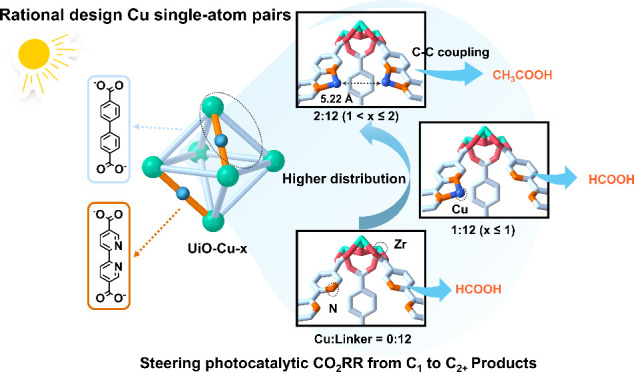

## Introduction

Artificial photosynthesis, specifically the photocatalytic CO_2_ reduction reaction (CO_2_RR) to value-added multicarbon (C_2+_) products, has attracted significant and sustained research interest since Halmann’s groundbreaking demonstration in 1978.^1^ Mimicking natural photosynthesis, this approach offers a dual strategy for greenhouse gas mitigation and renewable fuel production by harnessing solar energy.^2^ While extensive efforts have focused on converting CO_2_ into C_2+_ chemicals, such as ethanol and acetic acid, low yields and insufficient control over product selectivity necessitate further advancements.^3^ Over the past few decades, significant strides have been made in improving CO_2_ conversion efficiency, approaching industrially relevant benchmarks. However, efforts to improve selectivity, especially toward C_2+_ chemicals, have been limited. The volume of recent studies focusing on C_1_ products poses a significant economic barrier to the widespread adoption of this technology.

Therefore, a central focus for advancement is enhancing selectivity toward C_2+_ products. The photocatalytic conversion of CO_2_ to C_2+_ products is inherently complex.^4^ Subtle variations in the molecular microenvironment, including the strategic incorporation of synergistic moieties to stabilize key intermediates, play a pivotal role in dictating reaction pathways. For instance, Yu et al. designed a crystalline carbon nitride that enables photocatalytic reduction of CO_2_ to CH_3_CH_2_OH, whereas its amorphous counterpart yields CH_4_. However, the ethanol selectivity remains low at 15.1% versus 47.3% for CH_4_, likely due to a lack of precise control over the proximity of active sites.^5^ While mixed-valence Cu_*x*_O species have also been shown to promote C–C coupling through in situ generated Cu^+^ centers,^6^ systematic investigations that precisely engineer the spatial distribution of such centers to elucidate their role in directing C_2+_ pathways remain scarce.

A prominent trend in catalysis involves the utilization of supported single-atom catalysts (SACs) to maximize atom efficiency for cost-effectiveness and enhanced sustainability.^7^ SACs, characterized by their site-specific activity across a variety of reactions, are a central focus of numerous atom-efficient catalytic processes. However, achieving high selectivity toward C_2+_ products in CO_2_RR remains a challenge for SACs, primarily due to the limited control over the microenvironment required for the concerted activation of *CO and subsequent complex reactions essential for C–C coupling.^8,9^ There are still ongoing debates on the molecular-level mechanisms by which local electronic structures are modified and specific intermediates/transition states for C_2+_ product formation are stabilized within this class of heterogeneous atomic catalysts.

More importantly, current studies on SACs for photocatalytic CO_2_RR to C_2_ products typically rely on the synergistic interaction between isolated single atoms and adjacent elemental sites within the host semiconductor. Such interactions often construct rigid [Metal site–*OC–CO*–Elemental site] bridges or related motifs that stabilize key intermediates, in which the single-atom active sites cooperate with neighboring elements to form asymmetric or symmetric dual/tri sites (e.g., Cu–In in In_2_O_3_/Cu–O_3_,^8^ N–Cu–N in Cu–CN,^10^ and Cu–W in Cu_1_/W_18_O_49_ ^11^) for selective C_2+_ product formation. Despite these advances, the synthesis of such materials often relies on less-controlled defect-engineering strategies, which yield diverse and nonuniform binding configurations between the SACs and the semiconductor support. This structural ambiguity creates a significant hurdle for achieving a well-defined molecular-level understanding. Consequently, it remains unclear how microenvironments are modulated and how specific transition states leading to C_2+_ products are stabilized on most heterogeneous catalysts.

In this study, we employed UiO-67, a Zr-based metal–organic framework (MOF), as a molecularly precise matrix for grafting atomically dispersed Cu sites. To enable site-specific coordination, we partially substituted the biphenyl-4,4′-dicarboxylic acid (H_2_bpdc) ligands (the original linker in UiO-67) with 2,2′-bipyridine-5,5′-dicarboxylic acid (H_2_bpydc), introducing nitrogen-rich pockets for bidentate Cu anchoring. By systematically modulating the site density from 0.67 wt % to 2.0 wt %, we identified a programmed spatial threshold that dictates a dramatic shift in CO_2_RR. At low Cu loadings, the framework functions in a mononuclear regime, predominantly yielding C_1_ products. Conversely, higher Cu concentrations favor C_2_ products, with UiO-Cu-2 achieving an exceptional acetic acid selectivity of ca. 90%. Temperature-resolved electron paramagnetic resonance (EPR) spectroscopy provides compelling evidence for this distribution-dependent proximity, capturing a unique magnetic feature that emerges as torsional linker motions are suppressed at 100 K. Through photoelectrochemical measurements and density functional theory (DFT) calculations, we delineate two distinct catalytic regimes: (i) isolated Cu sites that preferentially stabilize *COOH intermediate, and (ii) correlated single-atom pairs (CSAPs) where neighboring Cu···Cu motifs facilitate C–C coupling via a stabilized *OCCOH pathway. This work establishes a quantitative model that directly links Cu spatial distribution with C_2_ selectivity, offering fundamental insights into synergistic electronic cooperativity within confined spatial regimes and advancing the rational design of photocatalysts for multicarbon synthesis.

## Results and Discussion

### Characterization and Atomic Structure of UiO-Cu-*x*

The synthetic procedure for UiO-Cu-*x* is illustrated in Figure 1a. First, a UiO-67-bpy precursor was specifically programmed by substituting 16.7% of original H_2_bpdc linkers with bipyridine-containing H_2_bpydc linkers, corresponding to a fixed H_2_bpdc-to-H_2_bpydc molar ratio of 10:2. Copper atoms were then incorporated into the MOF via impregnation, yielding the UiO-Cu-*x* catalyst with the desired single-atom Cu configuration. Powder X-ray diffraction (PXRD) analysis (Figure S1–S6) confirmed that the structural integrity and high crystallinity of the samples were preserved after metalation. All synthesized samples exhibit a cubic lattice with the space group of *Fm* 3 *m*. The crystallographic parameters are summarized in Table S1 and S2. Notably, the lattice parameter remained nearly unchanged at *ca*. 26.8 Å, with a negligible fluctuation of ∼0.01% upon Cu incorporation, indicating that copper integration did not disrupt the crystal structure of UiO-67. Furthermore, the absence of additional Bragg peaks for elemental Cu or Cu oxides confirms that no crystalline aggregates formed during the synthesis.

**Figure 1:**
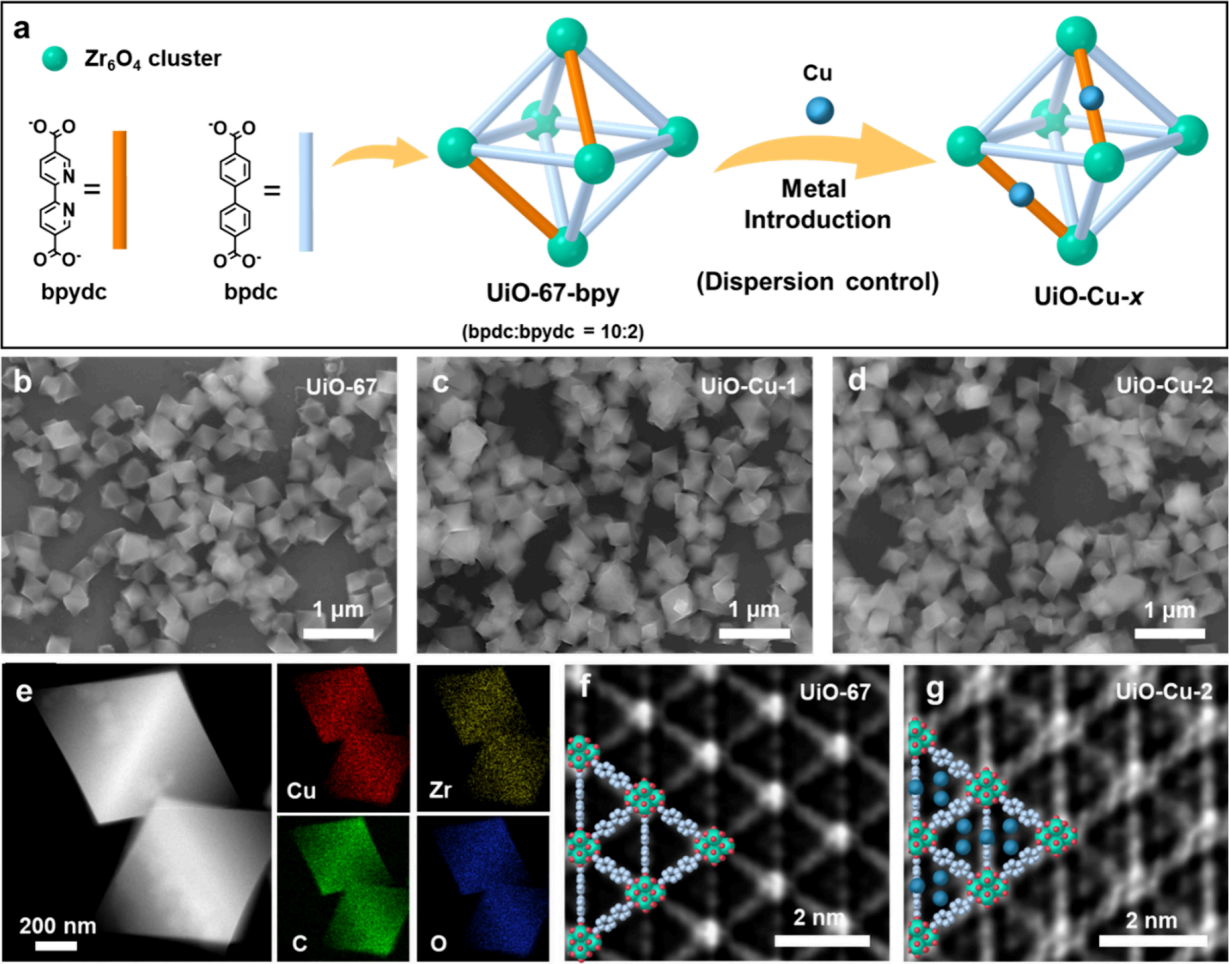
(a) Schematic illustration of the synthetic process for UiO-Cu-*x*. SEM images of (b) UiO-67, (c) UiO-Cu-1, and (d) UiO-Cu-2. (e) High-resolution HAADF-STEM image and EDX elemental mapping of UiO-Cu-2. Structural models and iDPC-STEM images of (f) UiO-67 and (g) UiO-Cu-2, viewed along the [110] crystal direction.

Scanning electron microscopy (SEM) further supports this finding, revealing that the nanocrystallites of the metalated samples retain a well-defined octahedral morphology (Figure 1b–d). Elemental analysis (Table S3) confirmed the copper atomic percentages in UiO-Cu-*x*, with the Cu:Zr atomic ratio increasing linearly from 0.048:1 to 0.088:1, 0.124:1, and 0.149:1 across the series (with *x* = 0.5, 1, 1.5, and 2). UiO-Cu-2 was selected as the primary focus for material characterization, as it best exemplifies the catalytic chemistry central to this study. High-angle annular dark-field scanning transmission electron microscopy (HAADF-STEM) was used to visualize copper atoms in UiO-Cu-*x*. Energy-dispersive X-ray spectroscopy (EDX) color mappings (Figure 1e) revealed a homogeneous copper distribution in UiO-Cu-2, confirming the atomic-level incorporation of copper within the MOF. To further probe the arrangement and interactions of copper, we utilized integrated differential phase contrast (iDPC) imaging, a technique that enables atomic-resolution visualization of beam-sensitive materials (Figure 1f–g, and S7–S9). When viewed along the [110] crystal direction, structural differences between pristine UiO-67 and UiO-Cu-1 or UiO-Cu-2 became evident. The Zr_6_O_4_ secondary building units in both samples exhibited no significant distortion or contrast differences, remaining well-organized. However, the linker morphology differed noticeably. In pristine UiO-67, the phenyl rings aligned parallel to [110] appeared linearly arranged, with distinguishable carbon atoms. In contrast, copper incorporation led to blurred and broadened phenyl rings, likely due to torsional strain in the Cu-complexed bpydc linkers. Additionally, bright atomic dots adjacent to the phenyl rings emerged, indicating that copper atoms are anchored to the bpydc linkers rather than on Zr_6_O_4_ nodes. Finally, N_2_ physisorption analysis (Table S4 and Figure S10–S11) confirms the preservation of the host framework’s structural integrity. While BET surface areas exhibited nonmonotonic fluctuations (1188–1437 m^2^/g), they remained consistently higher than that of pristine UiO-67, with negligible changes in pore size distribution, ensuring unobstructed diffusion of guest molecules through the MOF channels.

We used synchrotron X-ray absorption spectroscopy to probe the chemical coordination environment of the copper sites in UiO-Cu-*x*. The Cu K-edge X-ray absorption near-edge structure (XANES) spectra (Figure 2a) reveal that the pre-edge features of UiO-Cu-1 and UiO-Cu-2 align closely with the spectral signature of Cu­(II) species. Extended X-ray absorption fine structure (EXAFS) spectroscopy analysis (Figure 2b–2e) identifies a prominent peak at *ca*. 1.5 Å, attributable to Cu–N/O backscattering. Quantitative curve fitting (Table S5) confirms consistent Cu–N/O bond lengths of *ca*. 1.95 Å with a coordination number of *ca*. 4. We further employed wavelet transform (WT) analysis to resolve the correlation between k-space and R-space data (Figure 2f). The highest-intensity lobe at *k* = 6.5 Å^–1^ (*R* = 1.5 Å) corresponds to N/O coordination. We did not observe noticeable Cu–Cu backscattering feature (*k* > 6.5 Å^–1^) in both UiO-Cu-1 and UiO-Cu-2, suggesting that the copper remains atomically dispersed, effectively ruling out the presence of metallic clusters or alloys. These results indicate that both materials share analogous coordination geometries, with copper atoms primarily bound to two nitrogen sites from the bpydc linkers. Given the similar electron densities of nitrogen and oxygen, the additional nitrogen atoms in the coordination sphere may also suggest interactions with hydroxyl groups during synthesis.^12^ Complementary thermogravimetric analysis excludes the presence of residual or physically adsorbed precursor species (Figure S12). We did not observe noticeable precursor-specific decomposition features; the samples maintain high thermal stability up to 300 °C. This suggests that the Cu centers are anchored by the nitrogen atoms of the bpydc linkers and axially saturated by two water or hydroxyl ligands. This conclusion is further supported by X-ray photoelectron spectroscopy (XPS), which corroborates the electronic and chemical states of copper in our samples (Figure S13–S14, Table S6).

**Figure 2:**
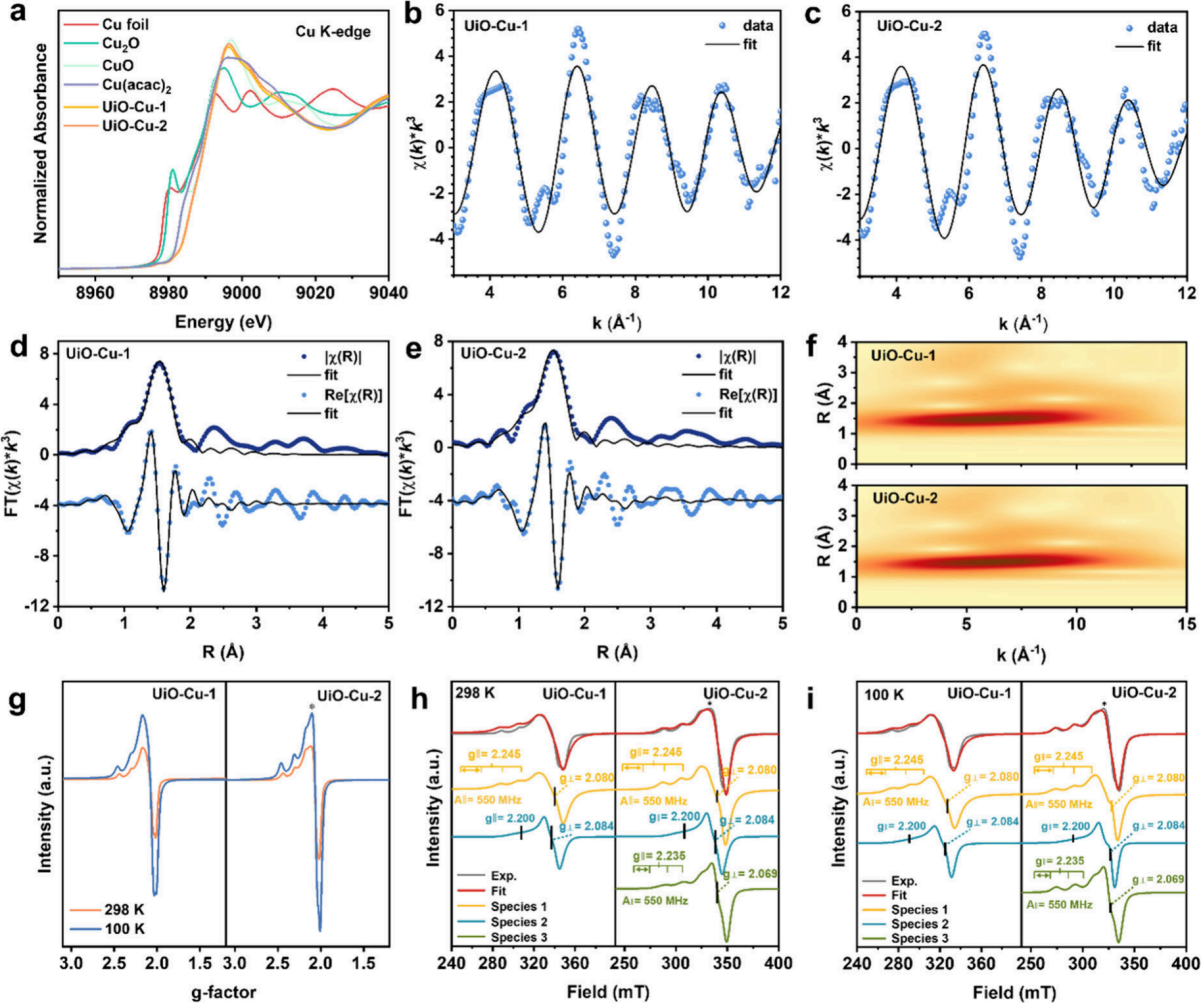
(a) Cu K-edge XANES spectra of Cu standards and UiO-Cu-*x* (*x* = 1 and 2). EXAFS spectra in k-space for (b) UiO-Cu-1 and (c) UiO-Cu-2. R-space representations (|χ­(R)| and Re­[χ­(R)]) for (d) UiO-Cu-1 and (e) UiO-Cu-2. X-band EPR spectra of the Cu species. Wavelet transform analysis of the Cu K-edge for (f) UiO-Cu-1 (top) and UiO-Cu-2 (bottom), with a Morlet wavelet with κ = 10 and σ = 1. (g) Temperature-dependent spectra of UiO-Cu-1 and UiO-Cu-2 at 298 and 100 K. The asterisk marks the additional feature present only in UiO-Cu-2. X-band EPR spectra of UiO-Cu-1 and UiO-Cu-2 with the best-fit simulation, data collected at (h) 298 K and (i) 100 K.

To probe the long-range spatial relationship between these dispersed Cu centers, we conducted temperature-dependent X-band EPR spectroscopy (Figure 2g–i, Table S7). At 298 K, both UiO-Cu-1 and UiO-Cu-2 exhibit typical Cu­(II) signals at (*g*_⊥_ = 2.080 and 2.084), assignable to the isolated Cu–N/O environments on the bpydc linker **(Species 1 (Cu–N/O)** and **Species 2 (Cu–N/O)**).^13^ Crucially, a distinct resonance at *g*_⊥_ = 2.069 (**Species 3**) emerges exclusively in UiO-Cu-2. The intensity of **Species 3** is sensitive to temperature; cooling to 100 K selectively amplifies this signal, while **Species 1** and **2** remain unaffected (Figure 2i). This temperature dependence is characteristic of coupled spin systems, in which cryogenic temperatures suppress the torsional lattice vibrations of the bpydc linkers, ‘locking’ the Cu centers (on the bpydc linkers) into a proximal orientation that enables electronic interaction. The absence of **Species 3** in UiO-Cu-1, despite an identical local coordination sphere, reveals that this additional signal is an operative spectroscopic feature of electronically coupled Cu­(II) single-atom pairs.

The atomic configuration of the copper sites was resolved using high-resolution synchrotron PXRD combined with Rietveld refinement, following established protocols (as also done in our previous work^12,14^ ). While the Bragg peak positions remained largely unchanged, significant intensity variations, particularly at higher 2θ angles, were observed upon metalation (Figure 3a–c). These changes result from altered scattering factors following the incorporation of heavier copper atoms. Quantitative analysis of the synchrotron XRD patterns (Figure S15 and Table S8) confirms the homogeneity of the metalation process, as evidenced by the highly symmetrical Bragg peaks across all samples. The Rietveld-derived crystal structures (Figure 3) reveal that copper atoms coordinate with the bpydc linkers within the UiO-67 framework, as detailed in Table S9–S12. To minimize refinement uncertainty, the site occupancy factors for the Cu sites were restrained at 0.07 (UiO-Cu-1) and 0.15 (UiO-Cu-2), based on elemental analysis results (Table S3). To directly clarify the Cu spatial distribution in the UiO-67 domain, the Rietveld-derived crystal structures for UiO-Cu-1 and UiO-Cu-2 are shown in Figure 3e–f. However, these crystal structures show ‘average’ that cannot capture subtle differences arising solely from variations in Cu occupancy and the associated changes in Bragg reflection intensities. Figure 3g provides a schematic illustration to more clearly visualize the UiO-Cu-*x* structures at different copper concentrations and to conceptually illustrate the Cu spatial distribution.

**Figure 3:**
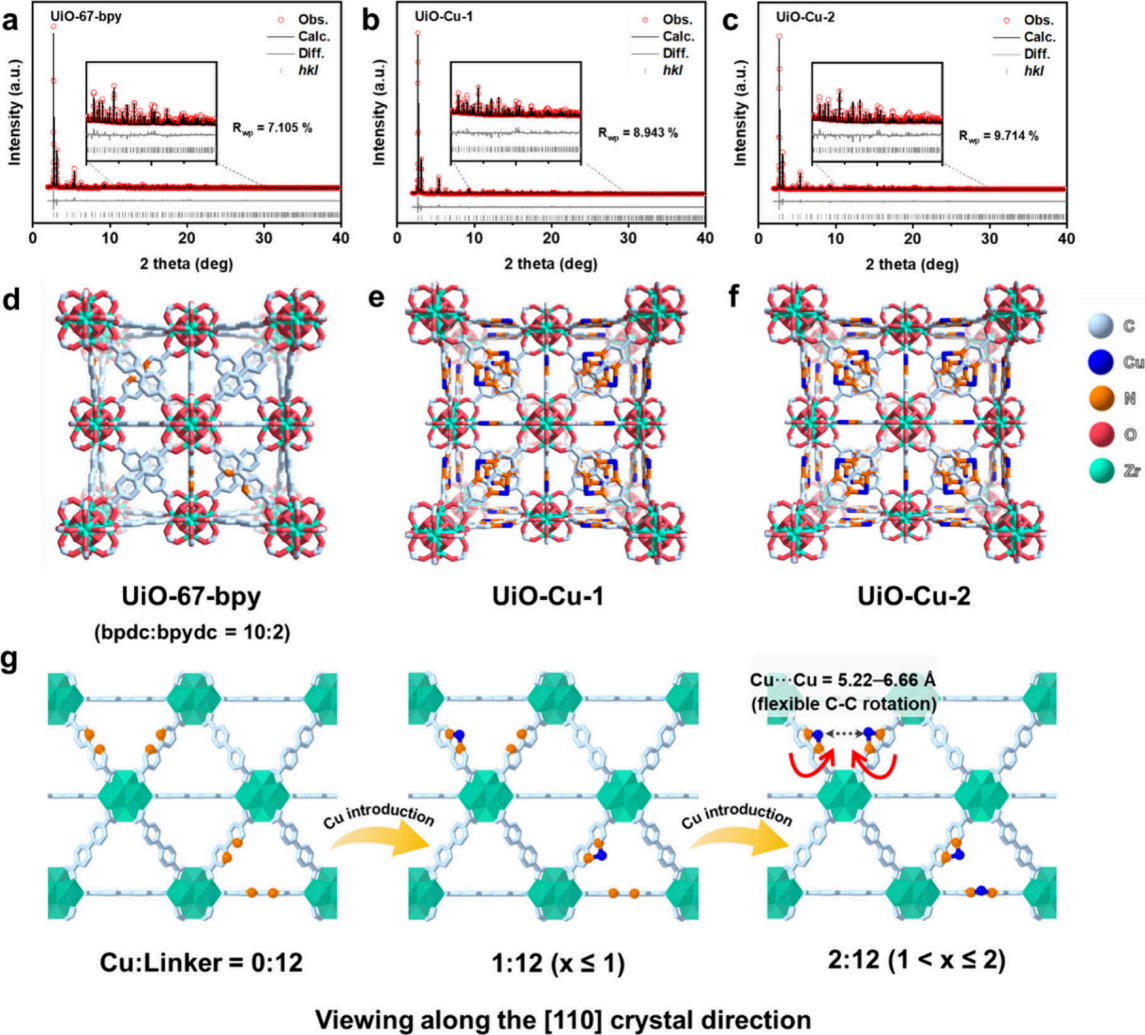
High-resolution synchrotron PXRD patterns (λ = 0.729589 Å) and the corresponding Rietveld refinement profiles for (a) UiO-67-bpy, (b) UiO-Cu-1, and (c) UiO-Cu-2. Rietveld-refined crystal structures for (d) UiO-67-bpy, (e) UiO-Cu-1, and (f) UiO-Cu-2. (g) Schematic illustration of copper distribution within the UiO domain, based on homogeneous and unbiased distribution of linkers and Cu sites.

The spatial distribution of the Cu sites is governed by the Cu-to-linker ratio, given homogeneous copper and linker dispersion from the Synchrotron PXRD characterizations. A structural formula unit of UiO-67 comprises [Zr_6_O_4_(OH)_4_ (bpdc)_5_(bpydc)]. By considering two adjacent structural units simultaneously, we can visualize the emergence of site proximity: at low Cu loadings (*x* ≤ 1), each unit pair can statistically accommodate at most one Cu atom (Cu:linker ≤ 1:12), ensuring isolated single-atom dispersion. As the loading increases (1 < *x* ≤ 2), each unit pair can hold at most two Cu atoms (Cu:linker ratio of 2:12), a high chance of forming ‘single-atom Cu pair’. Rietveld refinements of the synchrotron PXRD data provide a statistical interpretation of the atomic arrangement within the crystal structure. To reduce refinement uncertainty, the bpydc linkers were modeled in an orthogonal configuration (*θ*_*L‑L*_ = 90°), yielding a Cu···Cu distance of 6.66 Å. The Cu···Cu distance can reduce to 5.22 Å (*θ*_*L‑L*_ = 0°) due to the rotational flexibility of the bpydc linkers around the central C–C bond (Figure S16). This interatomic proximity is consistent with our findings observed in our temperature-dependent EPR spectra (**Species 3**).

### Photocatalytic CO_2_RR Performance

Following structural validation of the Cu site distribution, we evaluated the photocatalytic CO_2_RR activity of the UiO-Cu-*x* series in CO_2_-saturated deionized water with triethanolamine (TEOA) as a sacrificial hole scavenger. As shown in Figure 4a and Table S13, the pristine UiO-67 and UiO-67-bpy primarily facilitated the 2e^–^ reduction to HCOOH (41.5 and 14.7 μmol·g^–1^·h^–1^, respectively), with negligible CO generation. At low Cu loadings (*x* ≤ 1), where Cu centers are isolated, HCOOH remained as the dominant product. However, a sharp selectivity pivot toward acetic acid (CH_3_COOH) was observed as the Cu loading crossed the programmed spatial threshold. While UiO-Cu-1.5 achieved CH_3_COOH production at 62% selectivity, UiO-Cu-2 reached a rate of 48.6 μmol·g^–1^·h^–1^ with an 89% selectivity. This nonlinear transition from C_1_ to C_2_ products underscores the critical role of a specific copper site distribution. Beyond this concentration threshold, the increased probability of forming CSAPs effectively steers the reaction toward high-value C_2_ oxygenates.^15^ We have also benchmarked the performance of UiO-Cu-2 against state-of-the-art systems in Table S14, highlighting its superior selectivity for C_2_ oxygenates.

**Figure 4:**
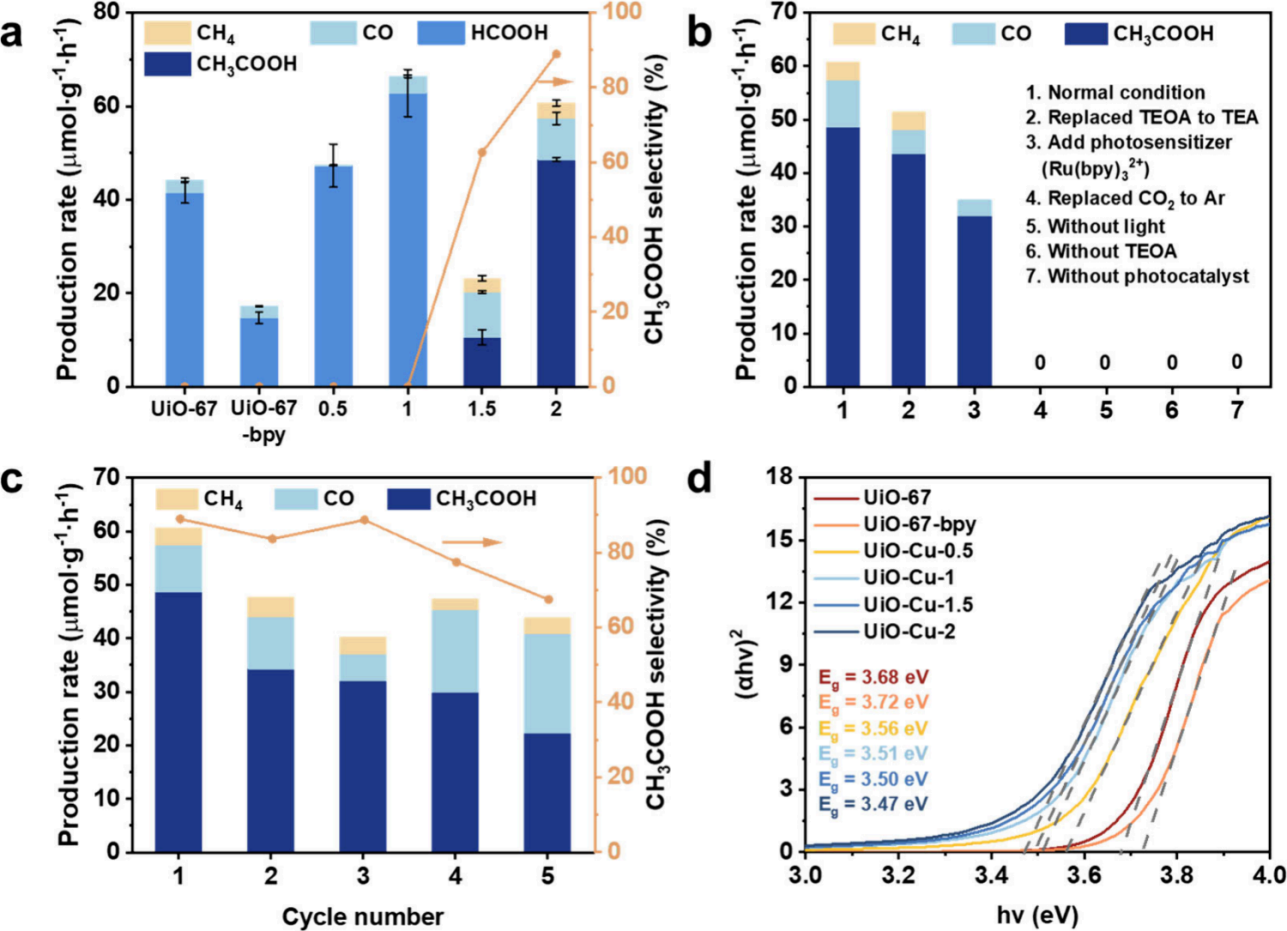
(a) CO_2_RR performance of UiO-67, UiO-67-bpy, and UiO-Cu-*x* under Xe light illumination using TEOA as the sacrificial agent. (b) Control experiments conducted under different reaction conditions. (c) Photocatalytic stability assessment of UiO-Cu-2. (d) Tauc plots of (αhν)^2^ vs photon energy for UiO-67, UiO-67-bpy, and UiO-Cu-*x* (*x* = 0.5, 1, 1.5, and 2).

To validate the photocatalytic mechanism and ensure the observed activity originates from the designed framework, a comprehensive series of control experiments was conducted (Figure 4b, S17–S19). The absolute requirement for CO_2_, TEOA, photocatalyst, and light was confirmed by the absence of activity when any single component was omitted. The use of triethylamine (TEA) as an alternative scavenger, along with ^13^CO_2_ isotopic labeling (Figure S17–S18), confirmed CO_2_ as the exclusive carbon source for CH_3_COOH. Additionally, introducing a photosensitizer (Ru­(bpy)_3_^2+^) did not enhance performance, likely due to inefficient electron transfer at the photosensitizer-MOF interface, or steric hindrance limiting access to active sites within the MOF pores. The heterogeneous nature of the catalysis was rigorously verified via a hot filtration test, which yielded a null result for postfiltration activity, thereby ruling out contributions from the leached species (Table S15). While UiO-Cu-2 retained approximately 75% of its initial CH_3_COOH yield after five consecutive cycles (Figure 4c), the post-mortem characterization reveals that this partial deactivation stems from textural evolution rather than active site degradation. Although PXRD indicates a loss of long-range crystallinity (Figure S20), surface area analysis (Figure S21, Table S16) reveals this is a transition from a microporous framework to a disordered mesoporous architecture. Crucially, ICP, STEM-EDX and XAS analyses (Figure S22–S24, Table S17–S18) confirm that the Cu centers remain atomically dispersed and chemically coordinated without aggregation.

To unravel the electronic structure and charge-carrier dynamics of UiO-Cu-*x*, we systematically investigated their photophysical properties. UV–vis diffuse reflectance spectroscopy (DRS) reveals that pristine UiO-67 exhibits an absorption edge near 400 nm, which undergoes a slight red-shift upon Cu incorporation (Figure S25).^16,17^ The wavelength-dependent apparent quantum yield profiles (AQY, Figure S26) of UiO-Cu-2 largely match the optical absorption below 500 nm, demonstrating that the photocatalytic activity is driven by the intrinsic photoresponse of the framework. The low AQY observed in the 500–800 nm range, despite significant light harvesting, is attributed to ligand-to-metal charge transfer (LMCT) from bpydc to Cu. The LMCT transition is likely accompanied by rapid exciton quenching, rendering this specific absorption region catalytically unproductive.^16,18^

To further delineate the electronic landscape, we extrapolated the bandgaps using Kubelka–Munk transformations.^19^ As shown in Figure 4d, the energy structures across the series remain remarkably comparable. The bandgap of UiO-Cu-*x* samples (3.50 eV) shows only marginal narrowing compared to pristine UiO-67 (3.68 eV). This minimal variance in bulk electronic parameters (3.47–3.56 eV) across the Cu-modified series is a critical finding; it demonstrates that the dramatic shift in C_2_ selectivity is not driven by bulk band-structure engineering but rather by local structural effects and the programmed proximity of Cu sites. Mott–Schottky analysis and computational valence band (VB) derivations (Figure S27, Table S19) further confirm that Cu incorporation only marginally perturbs the UiO-67 electronic landscape. The alignment of these band positions with the redox potentials required for acetic acid formation underscores the framework’s thermodynamic suitability, while the invariant electronic profile across *x* = 0.5 to 2.0 reinforces our conclusion that the CSAP threshold is the primary driver of C–C coupling.

To investigate the recombination dynamics of photogenerated electron–hole pairs, we employed steady-state and time-resolved photoluminescence (PL) spectroscopy. As shown in Figure 5a, pristine UiO-67 exhibits the highest PL intensity, characteristic of rapid radiative recombination. In contrast, the UiO-Cu-*x* series displays significantly quenched PL signals. This reduction signifies a suppressed recombination rate, attributable to efficient LMCT facilitated by the copper motifs anchored within the framework. Further insights into the carrier dynamics were obtained via time-resolved PL (TRPL) spectroscopy (Figure 5b). As illustrated in Figure 5b, UiO-Cu-*x* exhibits accelerated decay kinetics with effective lifetimes (τ_eff_) shortened to 1.000–1.069 ns, compared to 1.808 ns for UiO-67. Biexponential fitting (Table S20) reveals that the fast nonradiative decay component (τ_1_), dominated by trap-states mediated transfer, becomes the primary pathway in UiO-Cu-*x* (*A*_1_ = 85–88%). This is consistent with LMCT-driven exciton quenching, where the framework rigidity enables highly directional charge migration toward the Cu single-atom sites. This localized charge asymmetry creates a robust driving force for the spatial separation of carriers, ensuring that electrons are efficiently channeled to the surface active sites for CO_2_RR. The validity of this mechanism is further supported by photoelectrochemical measurements (Figure S28), where UiO-Cu-2 exhibits the highest photocurrent density. This provides compelling evidence that the programmed spatial distribution of Cu centers not only facilitates C–C coupling but also optimizes the fundamental charge-transfer efficiency of the UiO-67 host.

**Figure 5:**
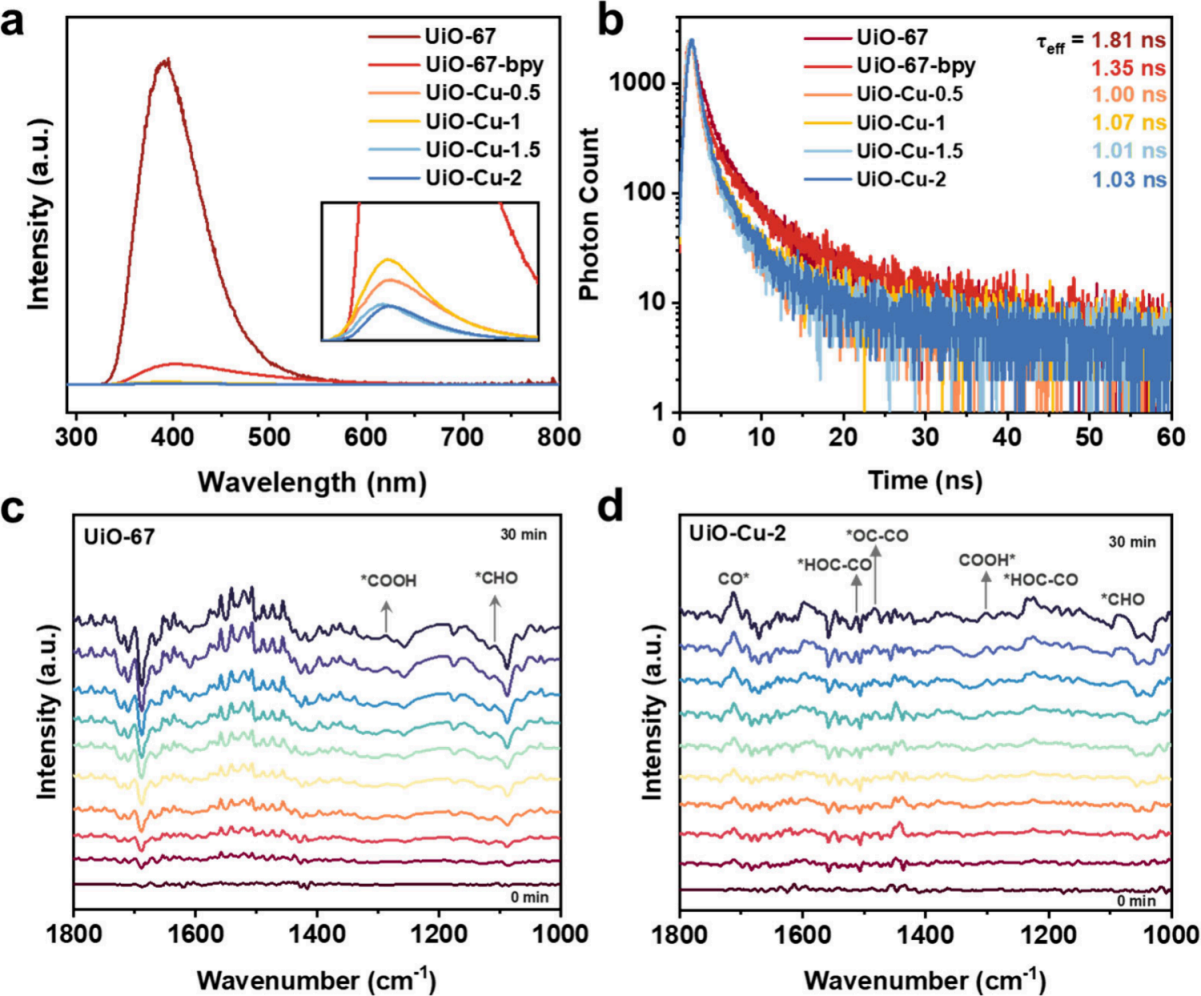
(a) PL spectra, (b) TRPL spectra of UiO-67, UiO-67-bpy, and UiO-Cu-*x*. *In-situ* DRIFTS spectra for CO_2_RR over (c) UiO-67 and (d) UiO-Cu-2.

Despite the significant improvements in photophysical properties observed by increasing copper loading, a fundamental question remains: Why does the product selectivity shift abruptly from C_1_ to C_2_ product? To address this, we employed *in situ* diffuse reflectance infrared Fourier transform spectroscopy (DRIFTS) to probe the surface-adsorbed intermediates under reaction conditions. The *in situ* DRIFTS spectra of UiO-67 (Figure 5c), UiO-Cu-1 (Figure S29), UiO-Cu-2 (Figure 5d), and reveal distinct spectral features. All samples exhibit IR bands at 1084 and 1302 cm^–1^, corresponding to *CHO and *COOH stretching vibrations,^20^ respectively, confirming the formation of C_1_ products. However, UiO-Cu-2 displays a unique IR band at 1714 cm^–1^, assigned to carbonyl stretching vibrations of *CO species (a configuration that is crucial for activating CO intermediates during photocatalytic CO_2_RR^21^). The emergence of this bridging *CO configuration is highly significant, indicating neighboring Cu···Cu motifs in UiO-Cu-2 (see Figure 3c) facilitate *CO binding more effectively, thereby providing a key mechanistic advantage for selective CO_2_ conversion. The absence of this peak in UiO-67 and UiO-Cu-1 suggests that isolated Cu sites are unable to stabilize such proximal intermediates. Furthermore, we observed key IR bands at 1480 cm^–1^ (*OC–CO) and 1225/1514 cm^–1^ (*HOC–CO), providing direct evidence for the stabilized dimer intermediates required for C–C coupling. Based on these observations, we propose a tailored reaction pathway over UiO-Cu-2: CO_2_ → M–CO* → *OC–CO → *HOC–CO.

To investigate the mechanistic origins of C_1_ and C_2_ product formation in CO_2_RR, we selected UiO-Cu-1 and UiO-Cu-2 as representative systems for DFT calculations. These samples correspond to one and two copper motifs per (C_82_H_52_O_32_Zr_6_N_2_)_2_, respectively, based on the statistical distribution analysis of copper discussed above. Prior studies have demonstrated that hydrogenation of CO_2_ either at the C or the O motifs can lead to the formation of C–H or C–O bonds, generating distinct intermediates: *OCHO and *OCOH.^22^ These intermediates play a pivotal role in forming *HCOOH and *CO products, respectively. Notably, *CO serves as a key coupling intermediate for the subsequent production of C_2_ products.^23^

As illustrated in Figure 6a, UiO-Cu-1 preferentially catalyzes CO_2_ hydrogenation at the carbon site, promoting the formation of HCOOH, which aligns with our experimental observations. In contrast, Figure 6b presents the complete reaction pathway for photocatalytic CO_2_RR over UiO-Cu-2, leading to the formation of CH_3_COOH. The potential-determining step (PDS) for the *HCOOH formation is *CO_2_ → *OCHO, with a potential barrier of 1.29 eV. For the CH_3_COOH pathway, the PDS is the *CO–*CO → *CO–*COH, which features a lower potential barrier of 0.95 eV. This reduced energy barrier indicates that the UiO-Cu-2 structure is more conducive to catalyzing CH_3_COOH formation via CO_2_RR. The comparison of reaction pathway energies underscores how copper concentration influences product selectivity between C_1_ and C_2+_ species.

**Figure 6:**
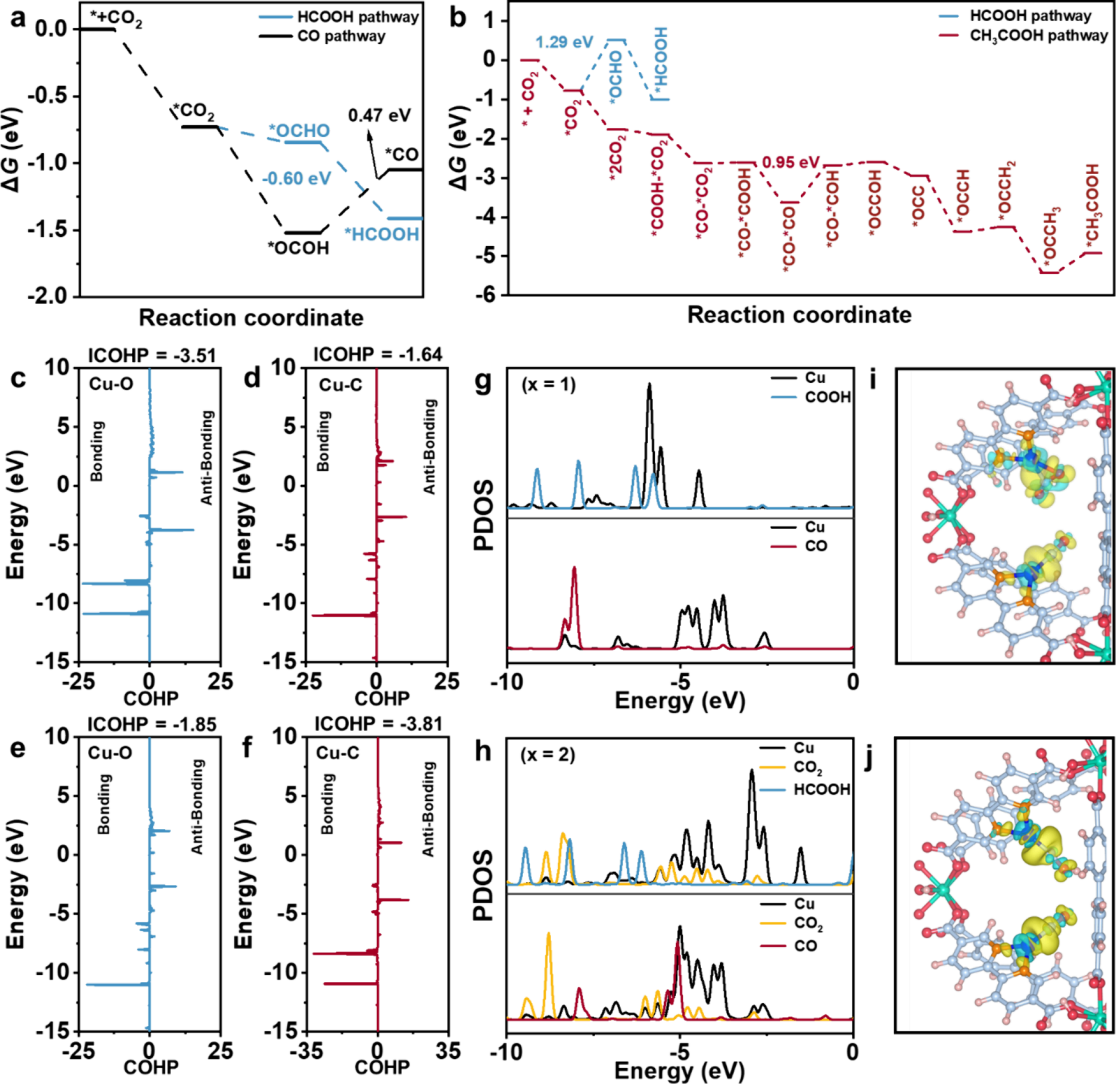
Gibbs free energy profiles for CO_2_RR steps over (a) UiO-Cu-1 and (b) UiO-Cu-2. COHP analysis of (c) Cu–O bond for *HCOOH, (d) Cu–C bond for *CO in UiO-Cu-1, (e) Cu–O bond for *HCOOH in *HCOOH–*CO_2_, and (f) Cu–C bond for *CO on *CO–*CO_2_ in UiO-Cu-2. PDOS analysis for (g) *HCOOH (top) and *CO (bottom) in UiO-Cu-1 as well as (f) *HCOOH–*CO_2_ (top) and *CO–*CO_2_ (bottom) in UiO-Cu-2. Charge density difference illustration for (i) *CO–*CO_2_ and (j) *CO–*COH, in UiO-Cu-2. The green area indicates a decrease in charge density, while the yellow area indicates an increase. Atoms are represented in the ball-and-stick model, with green, red, light blue, pink, and blue balls corresponding to Zr, O, C, H, and Cu, respectively. All DFT calculations were performed at the GGA/PBE level.

To elucidate the bonding interactions and electronic structure, we employed the crystal orbital Hamilton population (COHP) analysis to examine bonding/antibonding contributions, orbital participation, and bond strength variations.^24^ Our focus was on *HCOOH and *CO key intermediates in CO_2_RR over UiO-Cu-1, representing C_1_ products and the potential C_2_ products, respectively. As shown in Figure 6c, 6d, and S30, the integrated COHP (ICOHP) values reveal that the Cu–O bond in *HCOOH (ICOHP = – 3.55) is stronger than the Cu–C bond in *CO (ICOHP = – 1.66). This indicates that HCOOH adsorption is more favorable in UiO-Cu-1, promoting C_1_ product formation. For UiO-Cu-2, we analyzed two adsorption scenarios. Initially, we calculated the bond strengths for one Cu site occupied by either *HCOOH or *CO intermediate, while the neighboring site remains vacant (*□) (Figure S31, showing a single-site adsorption model). The ICOHP analysis revealed that the Cu–O bond in *HCOOH (ICOHP = – 3.55) is stronger than the Cu–C bond in *CO (ICOHP = – 1.66) (Figure S31c–d). This suggests that the concerted *CO adsorption at neighboring Cu sites is essential for stabilizing C_2_ precursors, a process that cannot occur at isolated sites.^25^ Consequently, we established a *HCOOH–*CO_2_ and *CO–*CO_2_ model (Figure S32, showing a dual-site adsorption model). In this configuration, when one Cu site is occupied by CO_2_, the neighboring Cu site exhibits stronger Cu–C bond characteristics compared to the Cu–O bond (Figure 6e–f). This indicates that the adsorption of CO_2_ on a Cu site enhances *CO adsorption at the neighboring Cu···Cu motifs in UiO-Cu-2 through a synergistic interaction.

We further analyzed the projected density of states (PDOS) to investigate synergistic cooperation between neighboring Cu···Cu motifs in UiO-Cu-2 (Figure 6g–h). For UiO-Cu-1 (single Cu site, Figure 6g), Cu exhibits greater orbital overlap with *HCOOH than with *CO. Conversely, for UiO-Cu-2 (Figure 6h), the PDOS reveals reduced overlap with *HCOOH orbital and enhanced overlap with *CO in the *HCOOH–*CO_2_ and *CO–*CO_2_ configurations. This confirms that adsorbed CO_2_ enhances the interaction between *CO and the Cu site, promoting *CO adsorption. It is noteworthy that *CO is a key coupling intermediate for the generation of C_2_ products as discussed above. The charge density difference (Figure 6i–j) for *CO–*CO_2_ and *CO–*COH on UiO-Cu-2 demonstrates significant electron transfer from CO_2_ to the Cu sites, increasing the electron density at the active sites. Both *CO and *COH intermediates exhibit electron-withdrawing behavior, promoting their adsorption and coupling, thereby enhancing the C_2_ product generation. The enhanced selectivity for C_2_ products arises not from a static Cu···Cu geometry but from electronic correlation between Cu sites. The rotational freedom of the bpydc linkers enables transient electronic interactions that stabilize bridging *CO species, thereby promoting C–C coupling.

By integrating *in situ* DRIFTS analysis and DFT calculations, we propose the following CO_2_RR pathway: Under light irradiation, UiO-Cu-*x* efficiently adsorbs CO_2_, activating it to form *CO_2_ species. Systems with higher copper distribution (1 < *x* ≤ 2) stabilize *CO_2_ more effectively than those with lower distribution (*x* ≤ 1), favoring its conversion to *COOH intermediates. The pathway progresses through key steps: *HOOC–*CO_2_, *OC–*CO_2_, *OC–*COOH, and *OC–*CO, ultimately driving the selective formation of CH_3_COOH. These results elucidate the mechanistic role of the distribution of single-atom copper in directing reaction pathways, emphasizing the importance of precisely engineered metal sites and their spatial distribution for optimizing C–C coupling efficiency and steering CO_2_ conversion toward value-added C_2+_ chemical products.

## Conclusions

In this study, we successfully engineered a series of supported single-atom copper sites with well-controlled site distribution within the UiO-67 framework, enabling the selective steering between single-carbon and multicarbon products from photocatalytic CO_2_RR. Our findings identify UiO-Cu-2 as a highly active and selective catalyst for acetic acid formation, demonstrating that copper site distribution plays a decisive role in determining product distribution. Through integrated experimental and theoretical analyses, we consistently show that optimal copper distribution facilitates C–C coupling and lowers the energy barrier of the PDS by modulating the local environment of UiO-67. Crucially, our results reveal that the selective formation of C_2_ products is governed by electronic coupling between copper sites which is a dynamic property distinct from static geometric arrangements. By demonstrating that site proximity is the prerequisite for initial C–C coupling, this work provides the essential first step in modulating the catalytic landscape. Once coupling is achieved via site cooperativity, specific kinetic tuning, either through metal identity or light energy, can then be used to steer the reaction toward diverse C_2_ targets. This principle offers a transferable strategy for designing SACs where spatial distribution and electronic communication are synergistically optimized, paving the way for next-generation catalysts in sustainable chemical synthesis.

## Methods

All the reagents were purchased from Dieckmann Company (Acros, Macklin, Alfa Aesar, and Thermo Scientific) without further purification.

### Synthesis of UiO-67

A mixture of 120 mg ZrCl_4_, 126 mg H_2_bpdc, and 1.88 g benzoic acid was prepared in a 100 mL beaker by adding 20 mL of a solvent composed of DMF and ethanol in a 4:1 volume ratio. The resulting solution was ultrasonicated for 30 min to achieve a homogeneous dispersion, then transferred to an autoclave and heated at 120 °C for 24 h. The suspension was subsequently washed three times with DMF and methanol, respectively. The final UiO-67 product was collected after being dried under vacuum.

### Synthesis of UiO-Cu-*x*

A mixture of 120 mg ZrCl_4_, 101 mg biphenyl-4,4′-dicarboxylic acid (H_2_bpdc, C_14_H_10_O_4_), and 21 mg 2,2′-bipyridine-5,5′-dicarboxylic acid (H_2_bpydc, C_12_H_8_N_2_O_4_) (with a molar ratio of H_2_bpdc to H_2_bpydc of 10:2) was combined with 1.88 g of benzoic acid and 20 mL of a solvent mixture (DMF to ethanol ratio of 4:1). The resulting mixture was ultrasonicated for 30 min to ensure proper dispersion, then transferred to an autoclave and heated at 120 °C for 24 h. Afterward, the suspension was washed three times with DMF and methanol, respectively. The UiO-67-bpy was collected following vacuum drying. Its theoretical molecular formula is C_82_H_50_O_32_Zr_6_N_2_, with a theoretical molecular weight of 2124.63 g/mol ([Zr_6_O_4_(OH)_4_(bpydc)­(bpdc)_5_]).

UiO-Cu was synthesized as follows: 1 mmol of UiO-67-bpy and a specified amount of copper­(II) acetylacetonate were mixed in 15 mL of methanol and then vigorously stirred at 55 °C for 24 h. The suspension was washed three times to remove any residual chemicals. The final UiO-Cu was collected after vacuum drying. The UiO-Cu samples were labeled UiO-Cu-x (where x = 0.5, 1, 1.5, 2), representing the theoretical molar ratio of Cu atoms to H_2_bpydc in UiO-Cu-*x*.

### Characterization

To determine the crystal structure and phase composition of the MOF, powder X-ray diffraction (PXRD) analysis was performed using a Rigaku SmartLab 9 kW-Advance system. For more detailed characterization, high-resolution synchrotron PXRD was conducted on BL02B2 at SPring-8, Japan. The incident X-ray flux had an energy of 16.99 keV and a wavelength of 0.729589 Å. A zero error of −0.00019(1)° was corrected using a high-quality standard CeO_2_ powder (NIST SRM674b). The as-prepared samples were encapsulated in 0.5 mm borosilicate capillaries and scanned using MYTHEN detectors. Data collection involved recording patterns within the 2θ range of 1.9–78° with a data binning of 0.006°. Each PXRD pattern was acquired for at least 10 min to ensure high-quality data with optimal contrast and angular resolution. It is important to note that the R-factors obtained using MYTHEN detectors are artificially elevated. Thus, the quality of the Rietveld refinement was assessed by examining the discrepancy between the fitted and observed data.

The obtained PXRD data were subsequently analyzed using the Rietveld refinement method with TOPAS v.6.0 software. Lattice parameters were determined, and the background was fitted using a Chebyshev polynomial with an average of 20 coefficients. The diffraction peaks were accurately modeled using the Thompson-Cox-Hastings (pseudo-Voigt) function. The final refined structural parameters for each histogram were obtained using the Rietveld method, incorporating fractional coordinates (*x*, *y*, *z*) and isotropic displacement factors (*B*_*eq*_) for all atoms. Additionally, the weighted profile factor (*R*_*wp*_) and goodness-of-fit values (*gof* = *R*_*wp*_/*R*_*exp*_) were calculated to assess the quality of fit, where *R*_*exp*_ represents the expected quality of the data.

Scanning electron microscopy (SEM) images were acquired using TESCAN VEGA3, and high-angle annular dark-field scanning transmission electron microscopy (HAADF-STEM) with energy dispersive X-ray analysis (EDX) imaging, and high-resolution iDPC-STEM images were conducted using a Cs-corrected STEM (Thermo Scientific Spectra 300 S/TEM) to investigate the surface morphology and assess particle size and elemental distribution within the MOF. All the data sets were acquired using a probe at 300 keV beam energy and 29.9 mrad probe-forming semiangle. The electron-beam current was reduced to <0.1 pA by an unfiltered monochromator. The dwell time was 2 μs, and the electron dose was <81 e^–^/Å^2^. The surface Cu oxidation states of the constituent elements were analyzed by X-ray photoelectron spectroscopy (XPS) using Thermo Scientific Nexsa spectrometer. Extended X-ray absorption fine structure (EXAFS) data were collected at Beamline TLS01C1 at Taiwan Light Source, employing a Si(111) double crystal monochromator to scan the photon energy. EPR spectra of the as-synthesized samples were obtained using a Bruker ELEXSYS-II E580 CW-EPR X-band spectrometer. The specific surface area and porosity of the as-prepared sample were investigated using nitrogen adsorption–desorption measurements conducted on a Micromeritics ASAP 2020 Micropore Analyzer. The optical properties of the as-prepared sample, including light absorption and bandgap energy, were studied using UV–visible spectroscopy (UV–vis) with PerkinElmer LAMBDA 1050, providing insights into the material’s photon absorption capabilities and its potential for facilitating photocatalytic processes. Photoluminescence spectroscopy (PL) and time-resolved PL (TRPL) measurements, conducted with an Edinburgh FLS 1000 Photoluminescence Spectrometer, were used to evaluate the luminescent properties of the MOF, aiding in understanding charge-carrier recombination processes and the fluorescent lifetime critical for efficient photocatalysis. The average lifetime (τ_eff_ = (A_1_τ_1_ + A_2_τ_2_)/(A_1_ + A_2_)) is extracted from the TRPL spectra by fitting with a biexponential function. The mechanism and intermediates of the photocatalytic CO_2_ reduction reaction were explored using *in situ* Fourier-transform infrared spectroscopy (DRIFTS) using Bruker Vertex-70 FTIR.

### Photoelectrochemical Properties

The photoelectrochemical properties were assessed using CHI760E set up in a standard three-electrode configuration. The electrolyte was 0.5 M Na2SO4, with an Ag/AgCl electrode as the reference and a platinum electrode as the counter electrode. For the preparation of the working electrodes, 10 mg of the sample was dispersed in 1 mL of a mixed solution with an anhydrous ethanol to Nafion volume ratio of 50:1. Then, 10 μL of the suspension was deposited onto a 1 × 1 cm^2^ SnO_2_:F (FTO) electrode, followed by solvent evaporation through heating at 150 °C for 2 h. A 300 W Xe lamp, providing an illumination intensity of 100 mW·cm^–2^, was used as the light source.

The conversion between potential versus the reversible hydrogen electrode (RHE) and versus Ag/AgCl was calculated using the following formula:

1 $$E_{RHE}=0.197+0.059^{*}pH+E_{Ag/AgCl}^{o}$$

The band gap of the as-prepared samples was calculated using the Kubelka–Munk equation, based on data obtained from UV–visible diffuse reflectance spectroscopy, as illustrated below.

2 $${\left(\alpha h\upsilon \right)}^{2}=A\left(h\upsilon -E_{g}\right)$$

where α, *h*, υ, *A*, and *E_g_* represent the absorption coefficient, Planck constant, frequency of light, constant, and band gap, respectively.

The conduction band (CB) was determined through Mott–Schottky (M-S) analysis conducted on an electrochemical workstation to measure the flat-band potential. The corresponding equations are presented as follows.

3 $$\frac{1}{C^{2}}=\frac{2}{\varepsilon \varepsilon_{0}eN_{D}}\left(E-E_{fb}-\frac{kT}{e}\right)\;\left(\mathrm{for n‐type semiconductor}\right)$$

where *C* represents the interfacial capacitance, ε is the relative dielectric constant, ε_0_ denotes the vacuum permittivity, *e* is the elementary charge, *N_D_* is the carrier concentration, *k* is the Boltzmann constant, *T* is the absolute room temperature, and *E_fb_* is the flat-band potential. According to the literature, UiO-67 is reported to be an n-type semiconductor.^26^ For n-type semiconductors, the flat-band potential is typically 0.1 to 0.3 V more positive than the conduction band potential.^27^

According to the following equation, the valence band potentials of the as-prepared sample were further obtained.

4 $$E_{VB}=E_{g}-E_{CB}$$

where *E_VB_* is the valence band potential, *E_g_* is the band gap, and *E_CB_* is the conduction band potential.

### Photocatalytic CO_2_ Reduction Reaction (CO_2_RR) Activity

The photocatalytic CO_2_ reduction reaction activity was evaluated using a closed reaction system. A 300 W Xe light source (GLORIA-X500A, Zolix Instruments Co., Ltd.) served as the illumination source. In a typical experimental procedure, 30 mg of the synthesized photocatalyst was added to a quartz reactor containing 50 mL of deionized water and 0.1 mL of triethanolamine (TEOA) as a sacrificial agent. Prior to initiating the reaction, the closed reaction system containing the solution was degassed for 30 min, followed by CO_2_ bubbling for an additional 30 min. This process was repeated to ensure the complete removal of residual gases from the system. The gaseous and liquid products were subsequently analyzed using a gas chromatography system (GC 2060, Shanghai Ruimin Instruments Co., Ltd.) and ^1^H NMR spectroscopy (Bruker Advance-III 400 MHz FT-NMR System), respectively.

The selectivity of acetic acid was calculated according to the following equation:

5 $$\mathrm{Selectivity of}\;{\mathrm{CH}}_{3}\mathrm{COOH}\left(\%\right)=\frac{\mathrm{moles of}\;{\mathrm{CH}}_{3}\mathrm{COOH}\times 2}{\mathrm{moles of total carbon of products}}$$

Based on the metal active sites in the catalyst, the turnover number (TON) was calculated using the following equations:

6 $$\mathrm{TON}=\frac{\mathrm{moles of total products}}{\mathrm{moles of active sites}}$$

While,

7 $$\mathrm{Moles of total carbon of products}=\mathrm{moles of CO}+{\mathrm{moles of CH}}_{4}+\mathrm{moles of HCOOH}+{\mathrm{moles of CH}}_{3}\mathrm{COOH}\times 2$$

### Calculation Methods

Density functional theory (DFT) calculations^28^ were carried out in the Vienna ab initio simulation package (VASP) based on the plane-wave basis sets with the projector augmented-wave method.^29^ The exchange-correlation potential was treated using the generalized gradient approximation (GGA) with the Perdew–Burke–Ernzerhof (PBE) parametrization.^30^ The van der Waals correction of Grimme’s DFT-D3 model was also used.^31^ To sample the k-space within the Brillouin zone, the Γ-centered Monkhorst–Pack grids were used^32^ along with a plane-wave cutoff energy of 450 eV. For structural optimizations, the atomic positions were allowed to relax in all three directions, while the shape and volume of the supercell were fixed until the convergence criteria for energy and force were set to 1 × 10^–5^ eV and 0.02 eV/Å, respectively.

## Supplementary Material



## Notes

Structural and Spectroscopic Characterizations: XRD patterns, STEM images, BET data, TGA curves, XPS spectra, synchrotron PXRD with Rietveld refinement analysis, FTIR, UV–vis, EXAFS, and crystal structure details; Product Analysis and Electrochemical Measurements: Gas chromatography and mass spectrometry (GC-MS) data, EIS results, electrochemical data, and apparent quantum yield (AQY); Catalytic Performance: CO_2_RR evaluation data for various catalysts; Computational Details: Additional discussion and data for DFT calculations (PDF)
