# Supplementary material for: Steering from C1 to C2 Products from the Photocatalytic CO2 Conversion over Correlated Single-Atom Pairs
Source: J Am Chem Soc. 2026 Jun 17;148(25):26865–77. doi: 10.1021/jacs.6c08783 (PMC13339133; doi:10.1021/jacs.6c08783)
Supplement: Supplementary file 1 [file ja6c08783_si_001.pdf]

# Supplementary Information

## Steering from $C_1$ to $C_2$ Products from the Photocatalytic $CO_2$ Conversion over Correlated Single-Atom Pairs

*Biyun Lin[1,#], Zixian Li[1,2,#], Zhen Zhan[3,#], Ka-Wa Wong[1], Yunong Li[1], Shogo Kawaguchi[4], Shintaro Kobayashi[4], Tai-Sing Wu[5], Wei-Min Tu[6], Yun-Liang Soo[6], Yufei Zhao[2], Songhua Cai[3,*], Jun Yin[3,7*], and Tsz Woon Benedict Lo[1,3,7,8*]*

*1 Department of Applied Biology and Chemical Technology, The Hong Kong Polytechnic University, Hung Hom, Hong Kong, 000000 China*

*2 State Key Laboratory of Chemical Resource Engineering, Beijing University of Chemical Technology, Beijing, 100029 China.*

*3 Department of Applied Physics, The Hong Kong Polytechnic University, Hung Hom, Hong Kong, 000000 China*

*4 Japan Synchrotron Radiation Research Institute (JASRI), 1-1-1 Kouto, Sayo-cho, Sayo-gun, Hyogo, 679-5198 Japan*

*5 National Synchrotron Radiation Research Center, 101 Hsien-Ann Road, Hsinchu, Taiwan, 30076 ROC*

*6 Department of Physics, National Tsing Hua University, Hsinchu 30013, Taiwan, 30013 ROC*

*7 PolyU-Daya Bay Technology and Innovation Research Institute, The Hong Kong Polytechnic University, Huizhou, Guangdong, 516081 China*

*8 The Hong Kong Polytechnic University Shenzhen Research Institute, The Hong Kong Polytechnic University, Shenzhen, 518057 China*

**Songhua Cai: Songhua.cai@polyu.edu.hk*

**Jun Yin: jun.yin@polyu.edu.hk*

**Tsz Woon Benedict Lo: benedict.tw.lo@polyu.edu.hk*

*[#] B.Y.L, Z.X.L and Z.Z contributed equally to this work.*

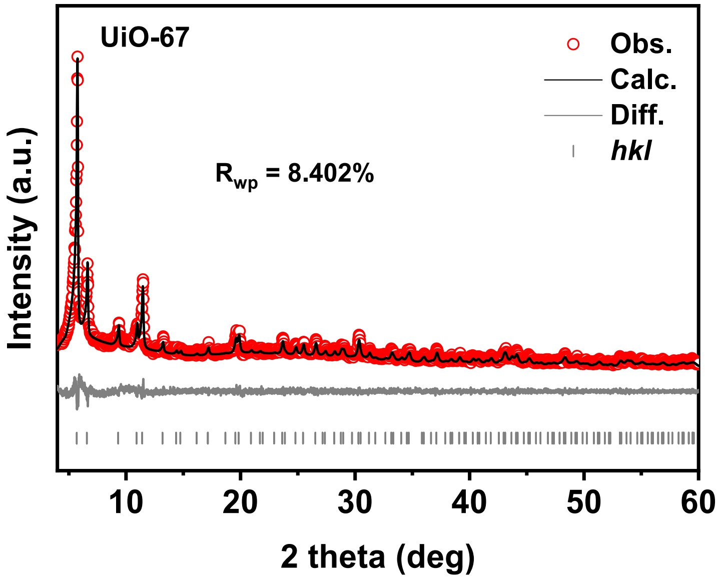


**Figure S1.** PXRD pattern and the Pawley refinement profile of UiO-67.

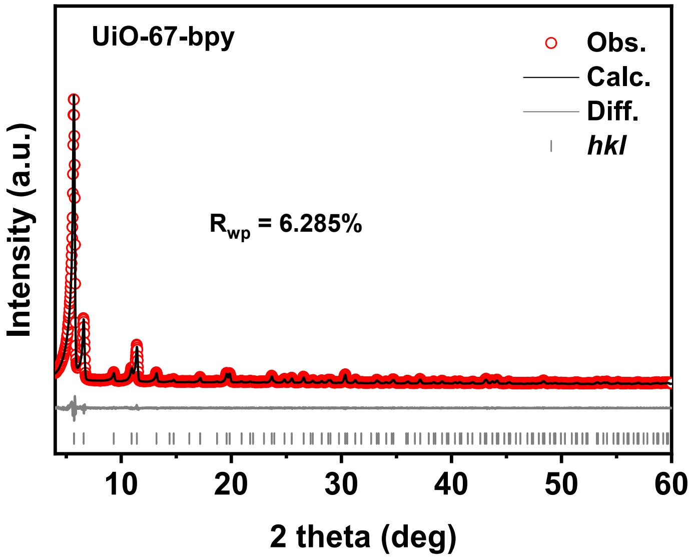


**Figure S2.** PXRD pattern and the Pawley refinement profile of UiO-67-bpy.

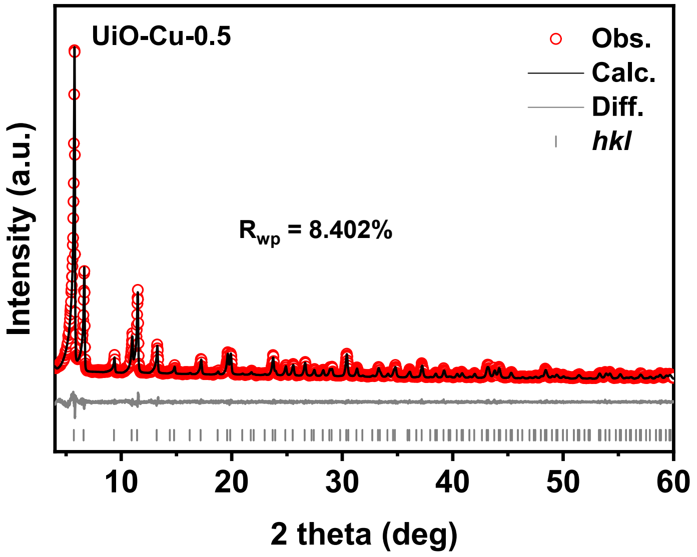


**Figure S3.** PXRD pattern and the Pawley refinement profile of UiO-Cu-0.5.

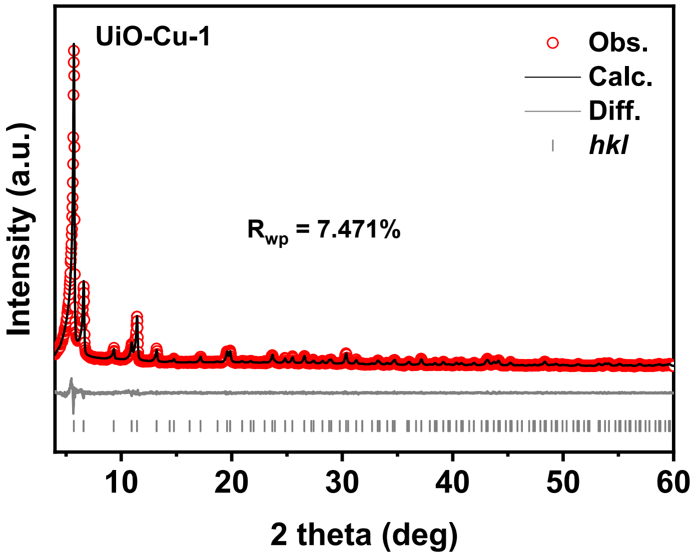


**Figure S4.** PXRD pattern and the Pawley refinement profile of UiO-Cu-1.

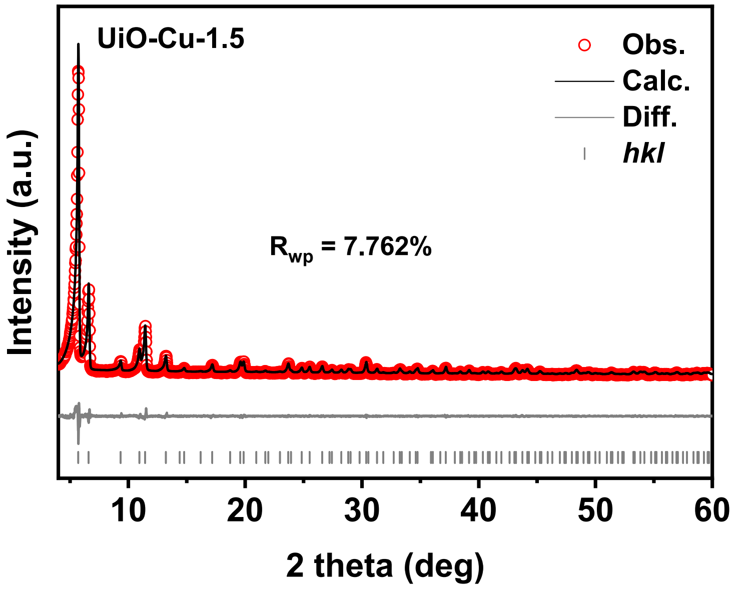


**Figure S5.** PXRD pattern and the Pawley refinement profile of UiO-Cu-1.5.

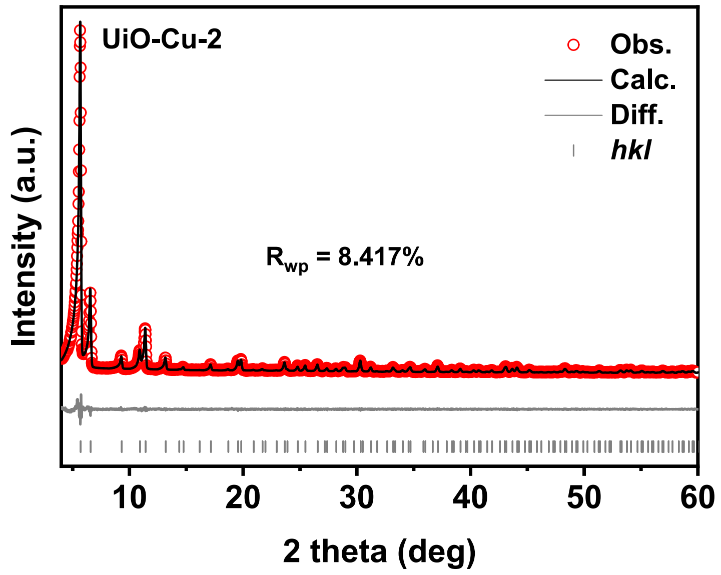


**Figure S6.** PXRD pattern and the Pawley refinement profile of UiO-Cu-2.

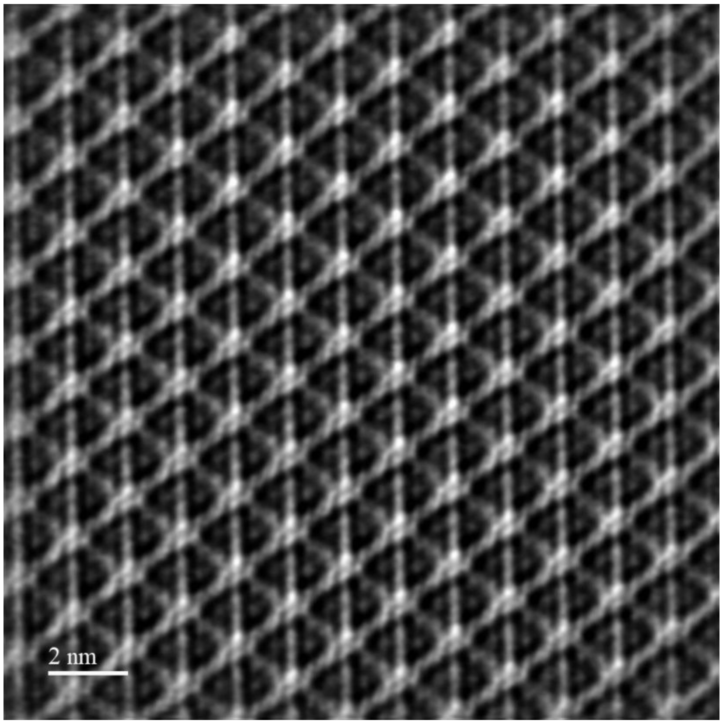


**Figure S7.** iDPC-STEM images of UiO-Cu-2, viewing along [110] crystal direction.

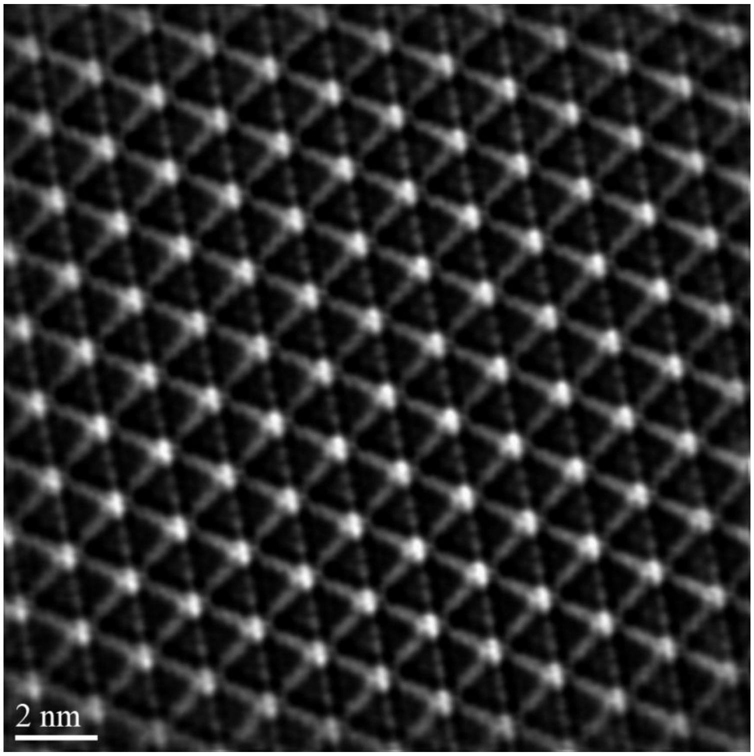


**Figure S8.** iDPC-STEM images of UiO-67, viewing along [110] crystal direction.

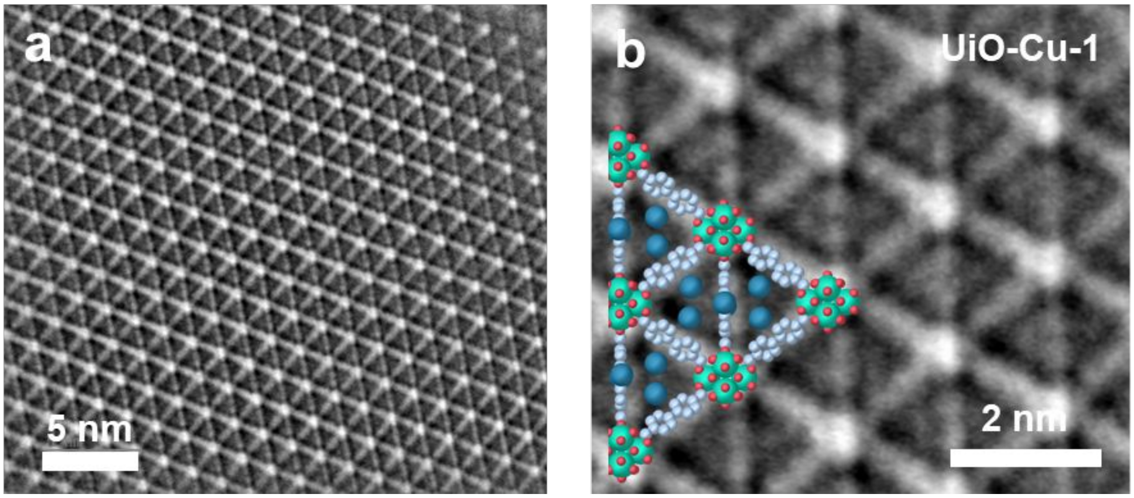


**Figure S9.** iDPC-STEM images of UiO-Cu-1, viewing along [110] crystal direction.

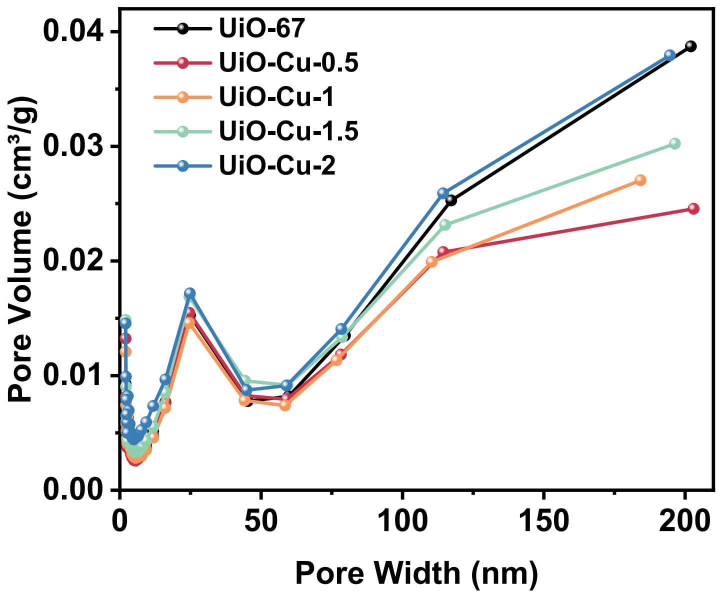


**Figure S10.** The pore size distribution results calculated from the BJH adsorption pore volume data of UiO-67 and UiO-Cu-*x*.

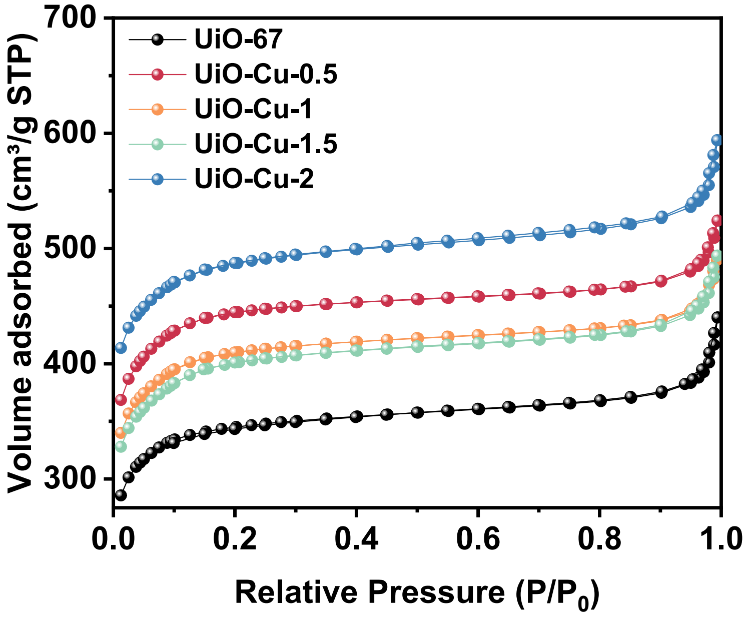


**Figure S11.** $N_2$ adsorption-desorption isotherm plots of UiO-67 and UiO-Cu-*x*.

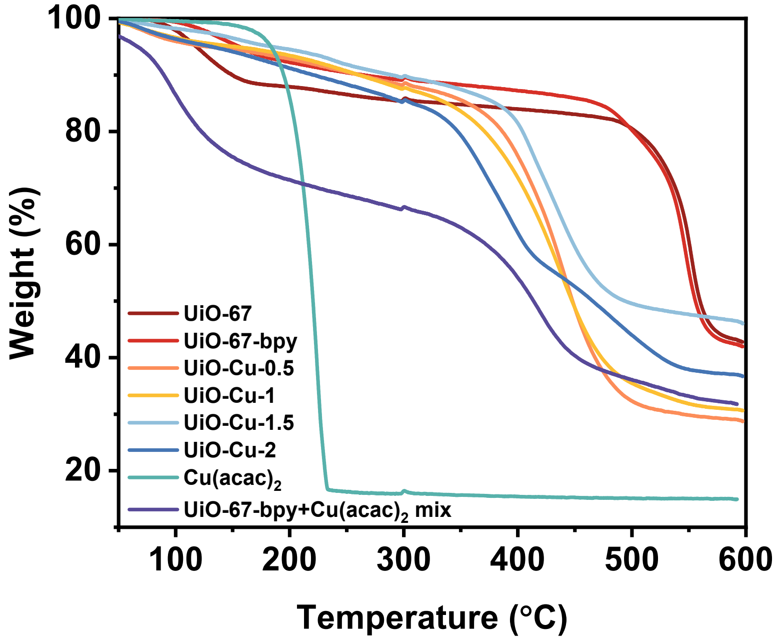


**Figure S12**. The TGA curve of UiO-67, UiO-67-bpy, UiO-Cu-*x*, $Cu(acac)_2$, and UiO-67 + $Cu(acac)_2$ mix.

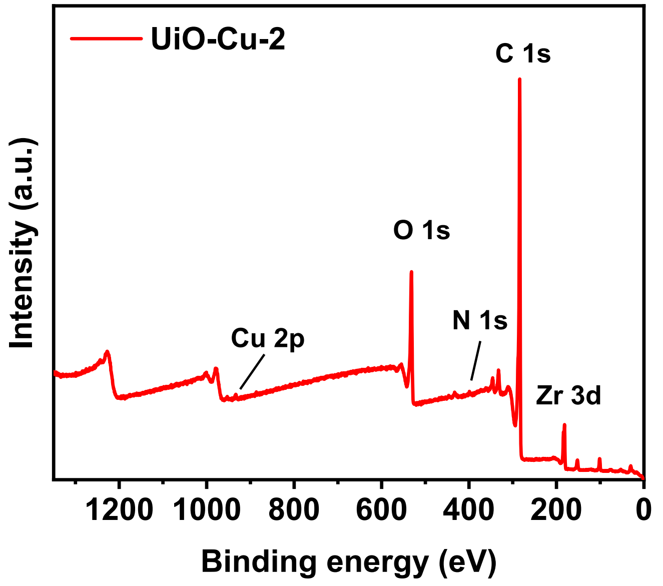


**Figure S13.** XPS full spectrum of UiO-Cu-2.

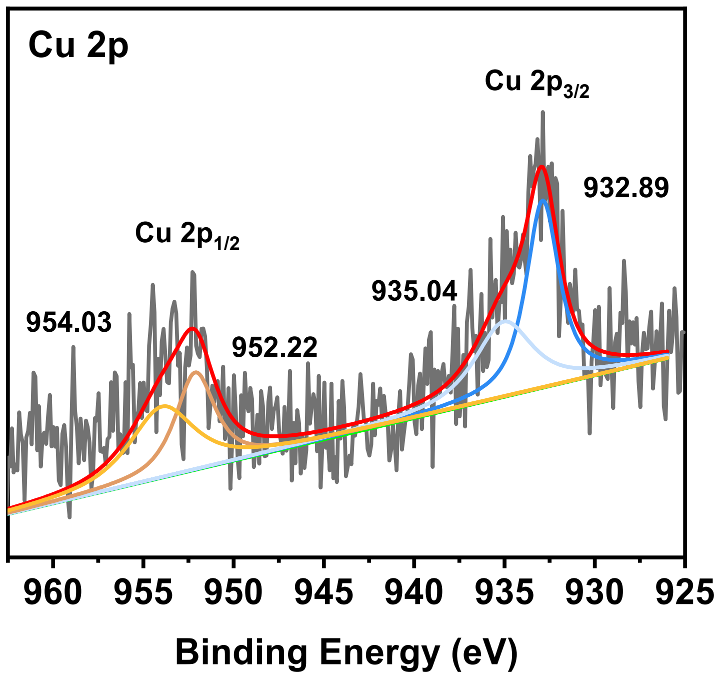


**Figure S14.** XPS spectrum of Cu 2p in UiO-Cu-2.

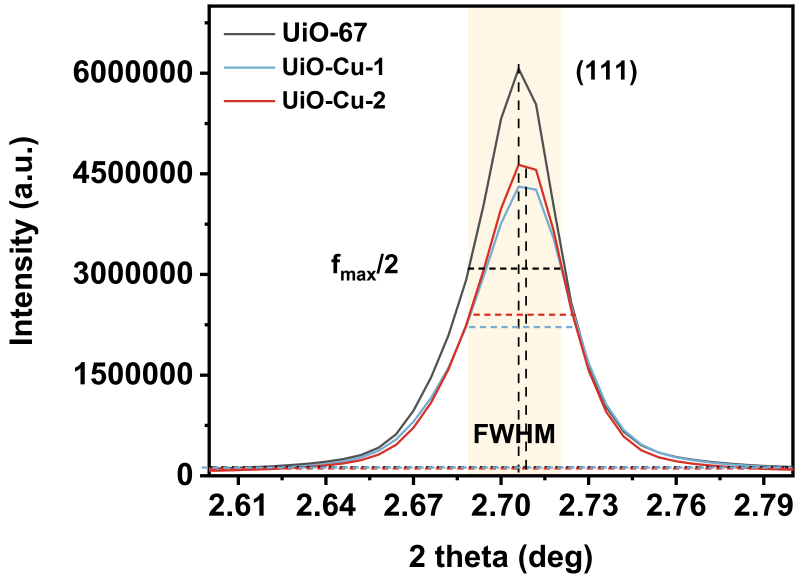


**Figure S15.** Comparison of the Bragg peak (111) over UiO-67, UiO-Cu-1, and UiO-Cu-2. The synchrotron PXRD data were collected on Beamline BL02B2 at SPring-8.

Considering the (111) reflection (2θ ≈ 2.71°) as a representative example, theoretical considerations suggest that if copper species were incorporated in a disordered fashion within the MOF structure, incoherent diffraction would arise. This would manifest as a shift in peak position and the emergence of asymmetric peaks. However, the Bragg peaks observed for UiO-67, UiO-Cu-1, and UiO-Cu-2exhibit symmetrical profiles (as indicated by asymmetry parameters of 0.0001 in **Table S6**), demonstrating a high degree of homogeneity within the samples. This observation implies that the metalation processes proceed uniformly throughout the MOF framework, resulting in a consistent distribution of copper active sites.

Further quantitative analysis of the synchrotron PXRD patterns (**Table S7**) yielded volume-weighted mean column heights (LVol) calculated from both the full width at half maximum (FWHM) and integral breadth (IB) values, assuming an intermediate crystallite size broadening described by a Voigt function. Similarly, the micro-strain, $e_0$, indicative of lattice imperfections

such as dislocations and vacancies, was derived from the FWHMs. The preservation of symmetry observed in UiO-67 (peak asymmetry parameter of 0.0001) following copper metalation in UiO-Cu-1 and UiO-Cu-2 reinforces the conclusion that copper sites are homogeneously and orderly dispersed within the MOF structure.

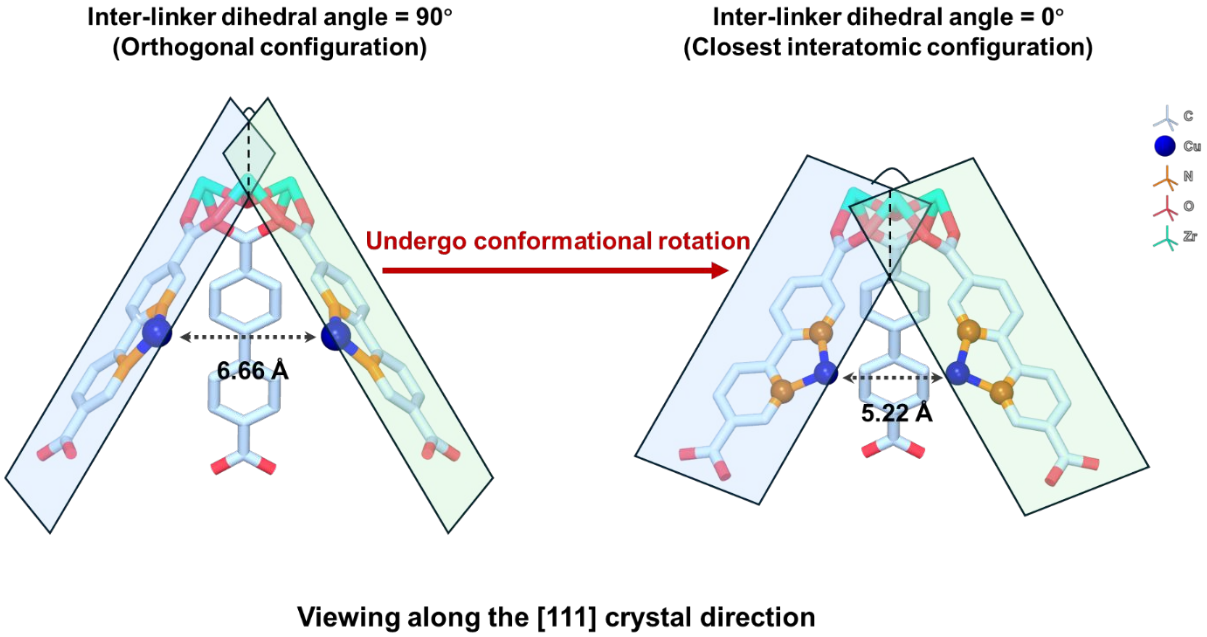


**Figure S16.** Schematic illustration of structural atomic distance in UiO-Cu-*x* viewed along the [111] direction.

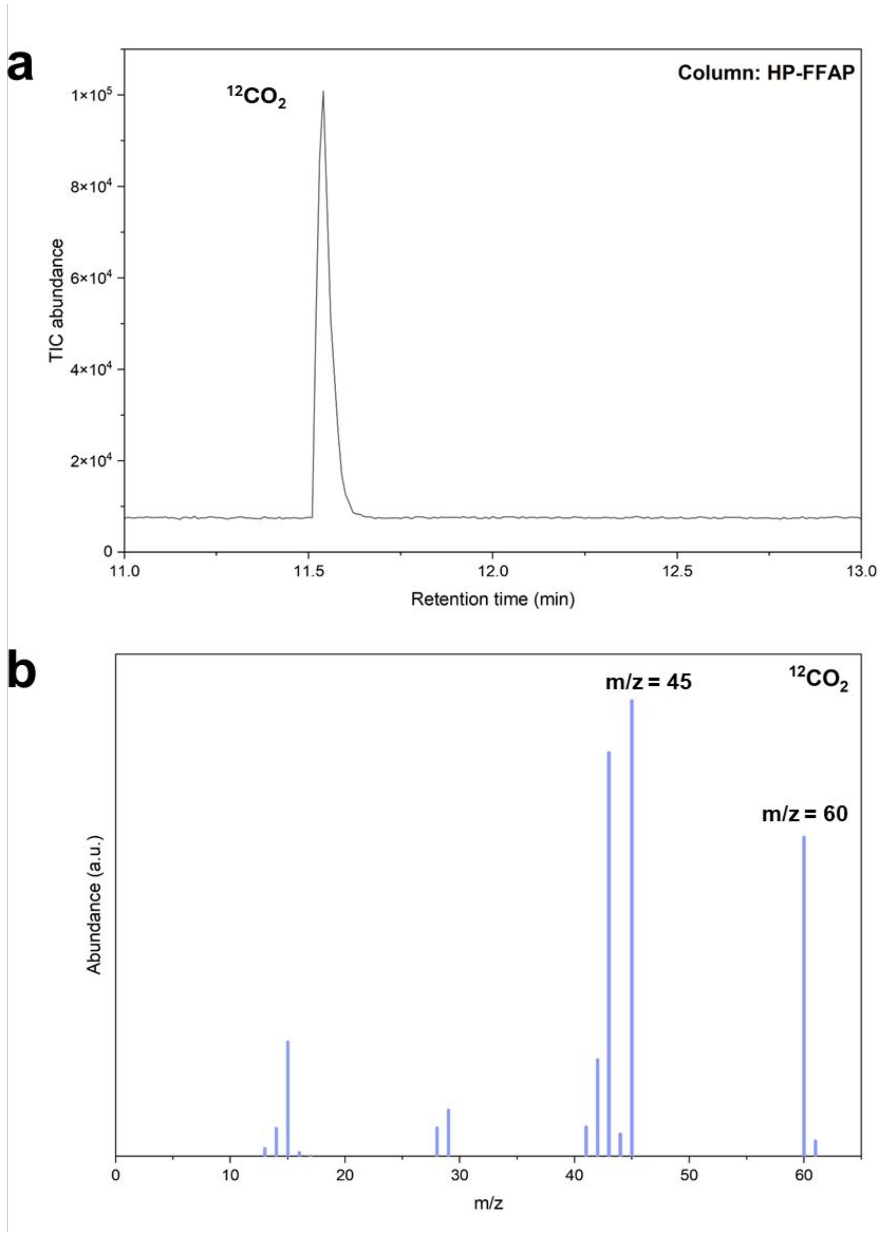


**Figure S17.** Gas chromatography and mass spectra analyses of the carbon source of the generated $CH_3COOH$ in the photochemical reduction of $^{12}CO_2$ by using UiO-Cu-2 as catalyst.

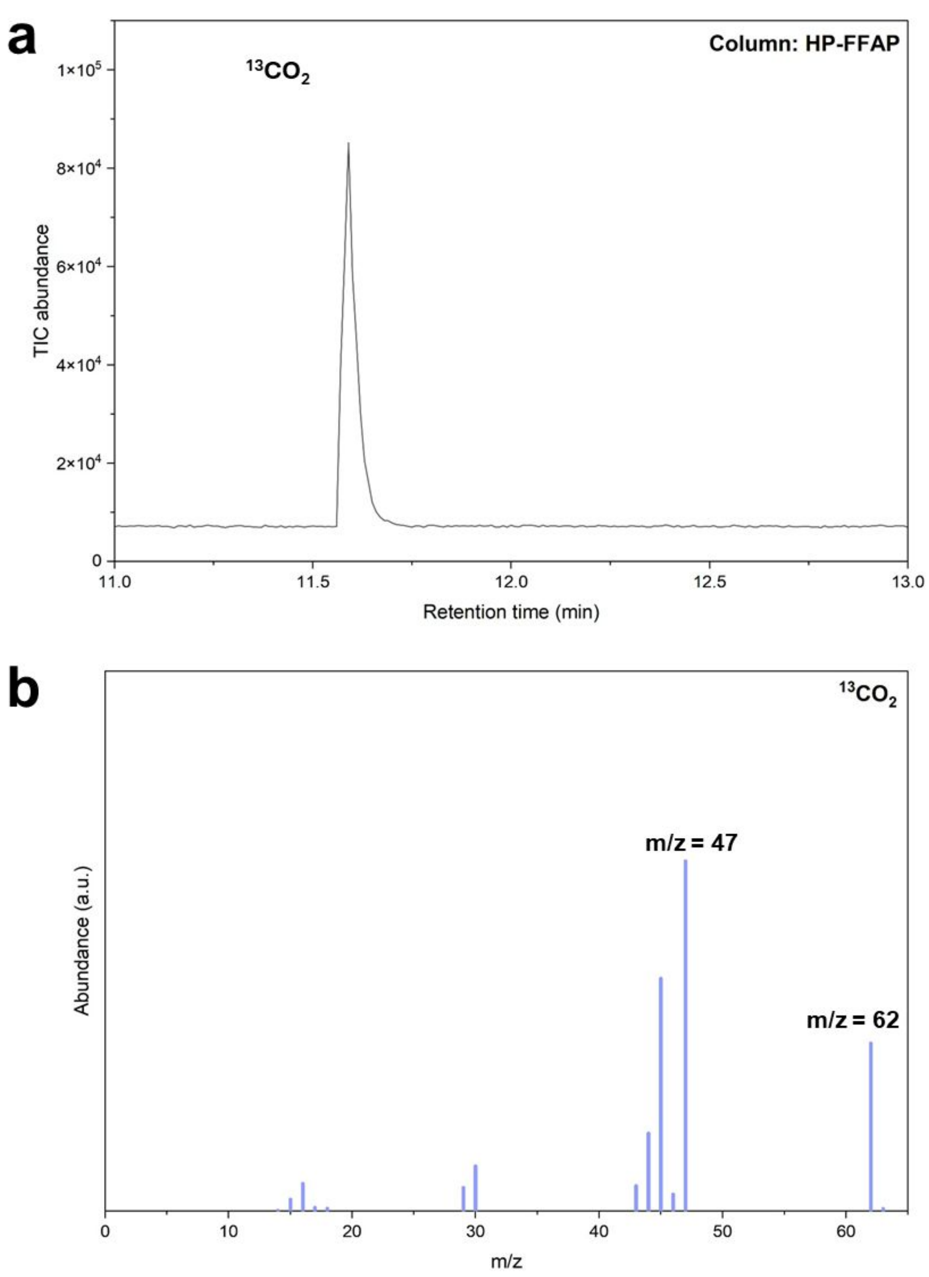


**Figure S18.** Gas chromatography and mass spectra analyses of the carbon source of the generated $CH_3COOH$ in the photochemical reduction of $^{13}CO_2$ by using UiO-Cu-2 as catalyst.

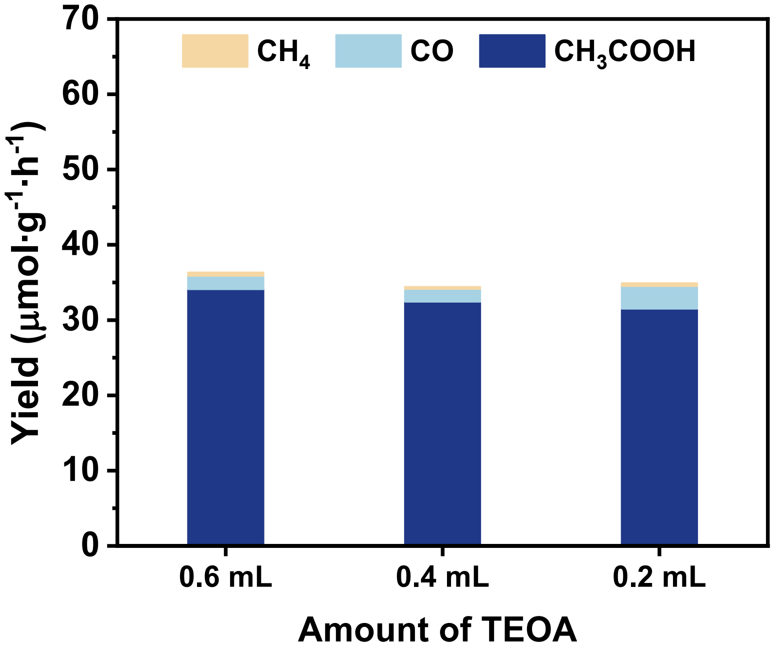


**Figure S19.** Control experiments under different amounts of TEOA for UiO-Cu-2.

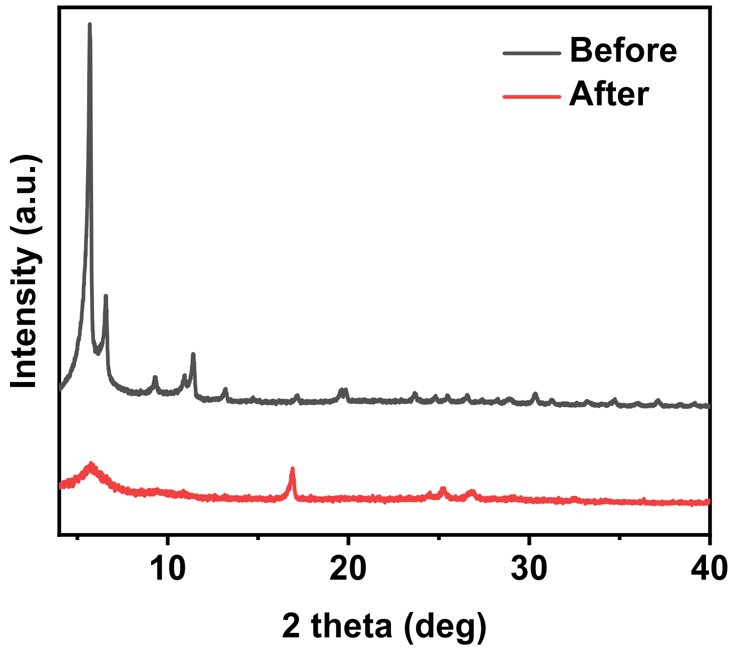


**Figure S20.** Comparison of the PXRD patterns of pristine UiO-Cu-2 and after five catalytic cycles.

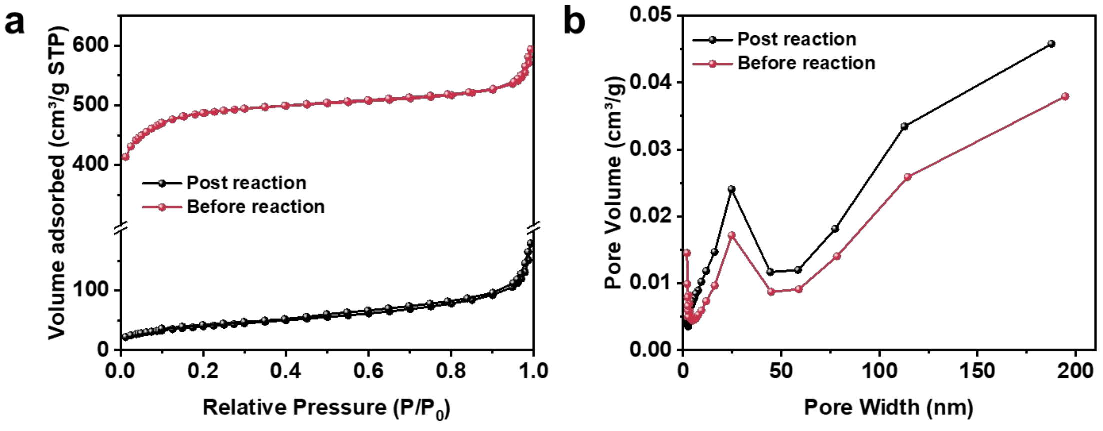


**Figure S21. (a)** $N_2$ adsorption-desorption isotherm plots and **(b)** the pore size distribution results calculated from the BJH adsorption pore volume data of UiO-Cu-2 before and after five catalytic cycles.

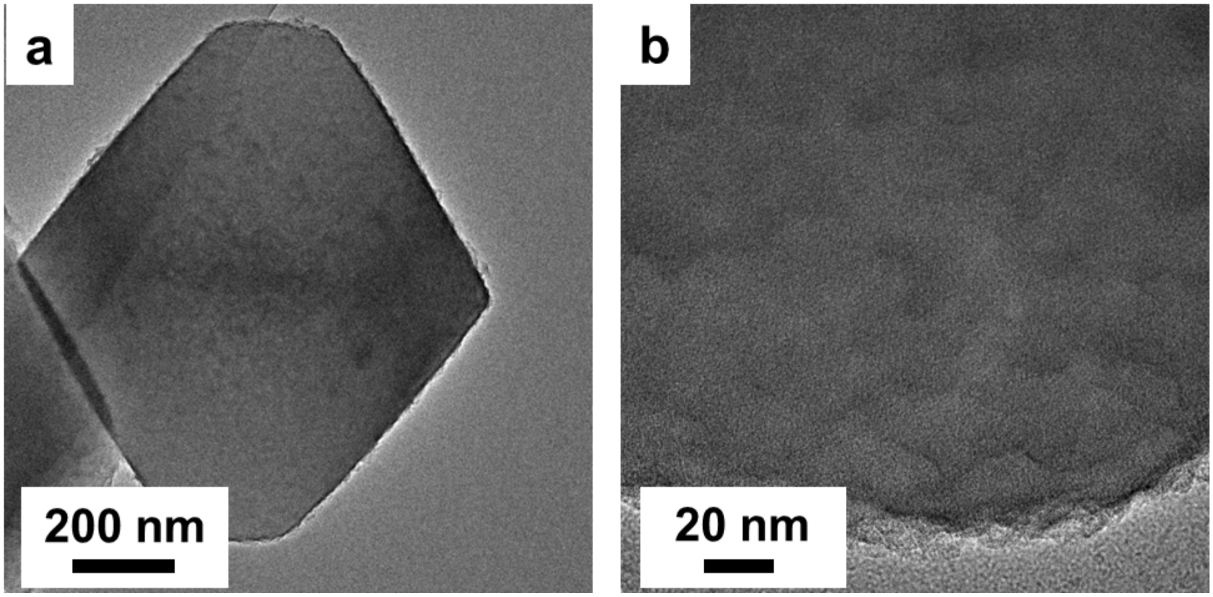


**Figure S22. (a, b)** TEM images of UiO-Cu-2 after five catalytic cycles.

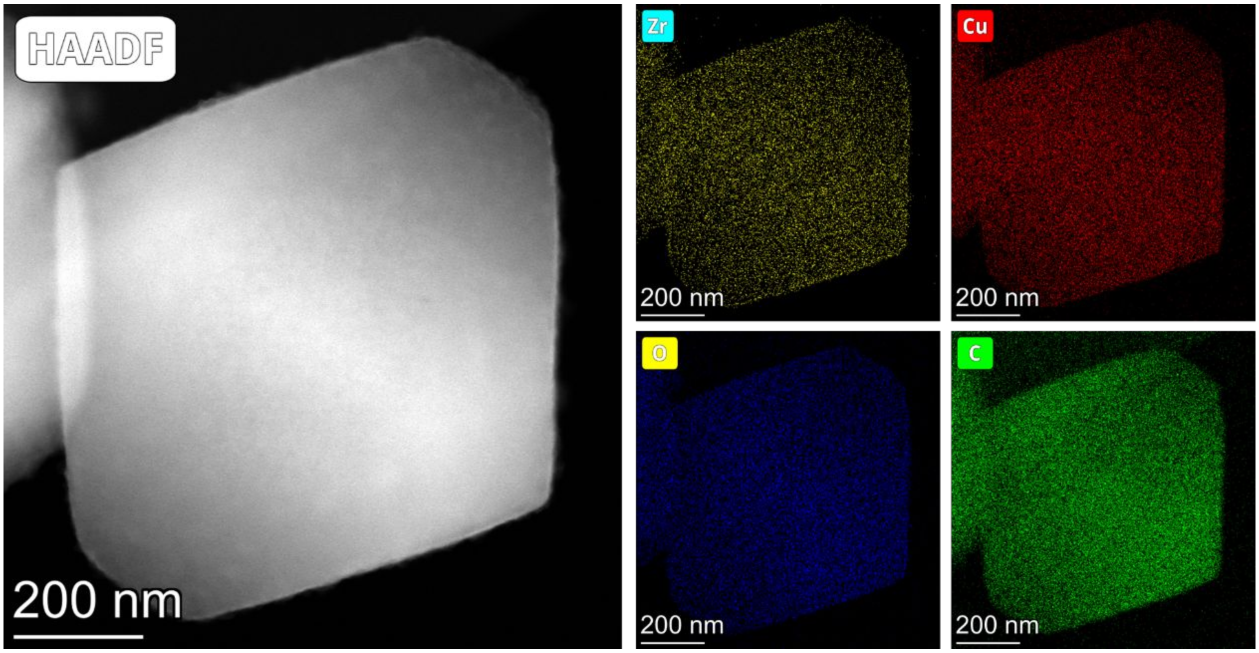


**Figure S23.** Corresponding EDX element mapping of UiO-Cu-2 after five catalytic cycles.

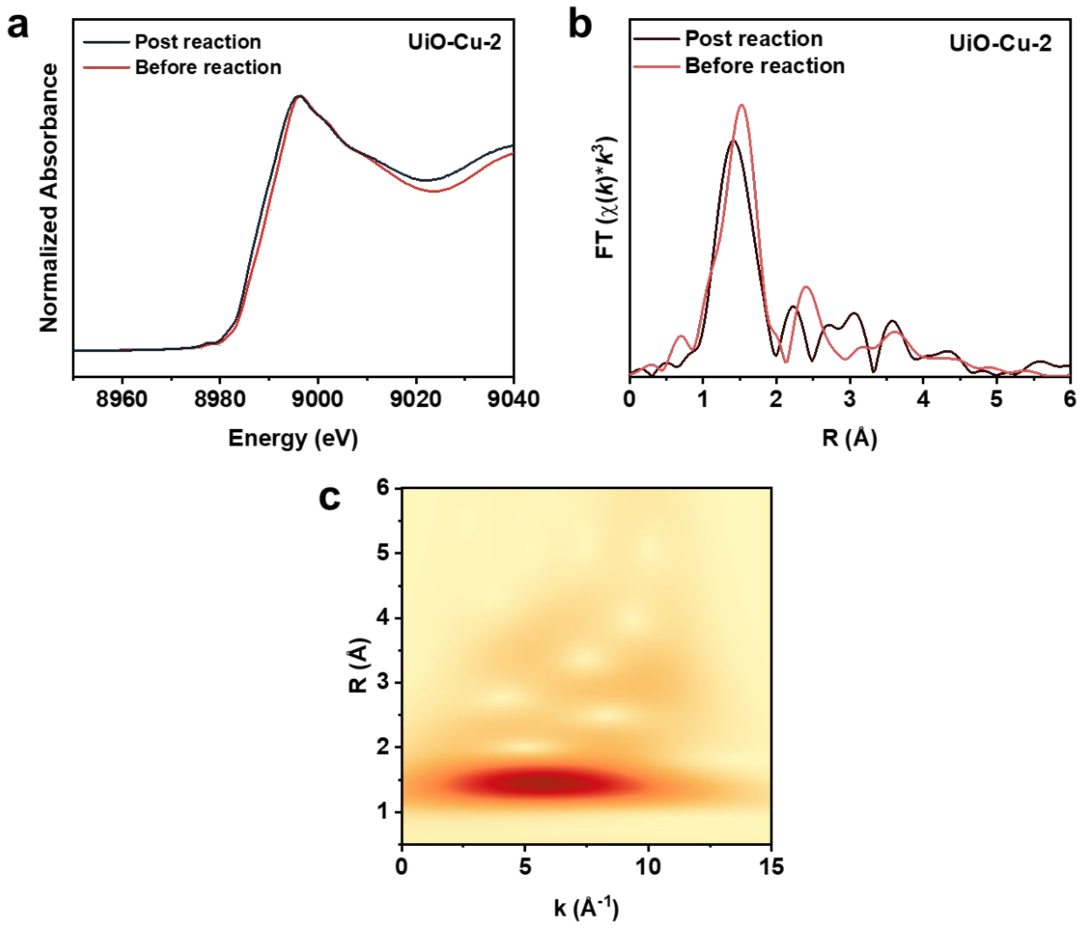


**Figure S24. (a)** Cu K-edge XANES spectra and **(b)** EXAFS spectra in R-space of UiO-Cu-2 before and after five catalytic cycles. **(c)** Wavelet transform analysis of the Cu K-edge for post-catalytic UiO-Cu-2.

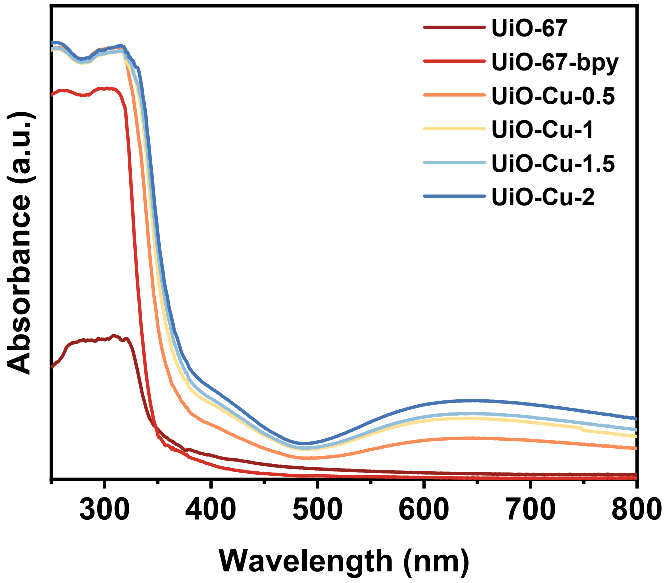


**Figure S25.** The UV-vis spectra of UiO-67, UiO-67-bpy, and UiO-Cu-*x* (where $x = 0.5$, 1, 1.5 and 2).

The apparent quantum yield (AQY) for $CO_2$ reduction was measured using 10 W LED lamp (PCX-50C Multi-Channel Photochemical Reaction System, PerfectLight) with different wavelengths (365, 385, 420 450, 485, 535, 595, 630, and 760 nm). The irradiation area was controlled as 1.5×1.5×π $cm^2$. The average intensity was determined by an optical power meter (TS560, Speedre). The AQY was calculated as follows equation:

$$AQY = \frac{N_e}{N_p} \times 100\% = \frac{10^9(v \times N_A \times K) \times (h \times c)}{(I \times A \times \lambda)}$$

Where, $N_e$ is the total number of transferred electrons, $N_p$ is the quantity of incident photon, $v$ is reaction rate ($mol{\cdot}s^{-1}$), $N_A$ is Avogadro constant (6.02×1023 $mol^{-1}$), K is Number of electrons transferred in the reaction (For acetic acid, K = 8), h is Planck constant ($6.626\times10^{-34}$ J·S), c is speed of light ($3\times10^8$ m/s), I is light intensity ($W{\cdot}m^{-2}$), A is the irradiation area ($m^2$), λ is the wavelength of light (nm).

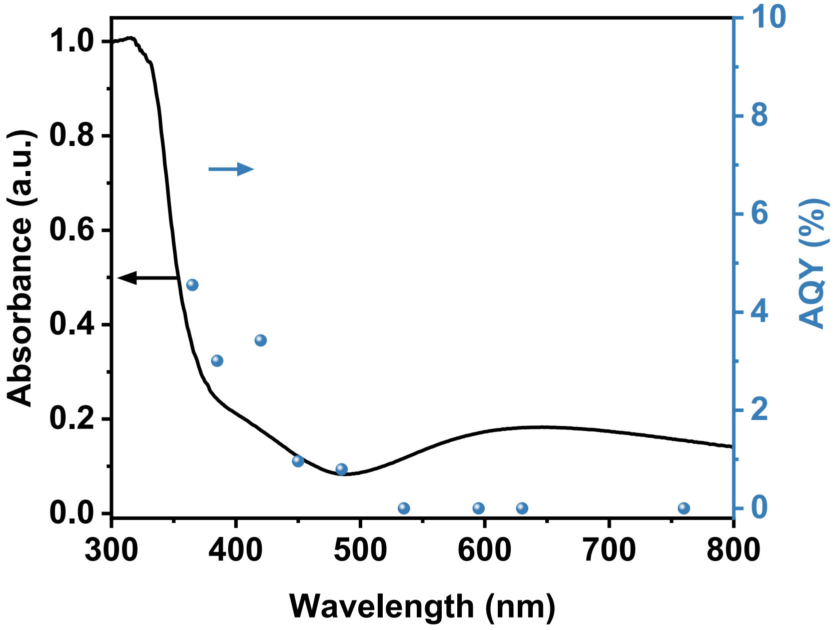


**Figure S26.** Wavelength-dependence of the AQY for photocatalytic $CO_2$ reduction and the UV-vis absorption spectrum of UiO-Cu-2.

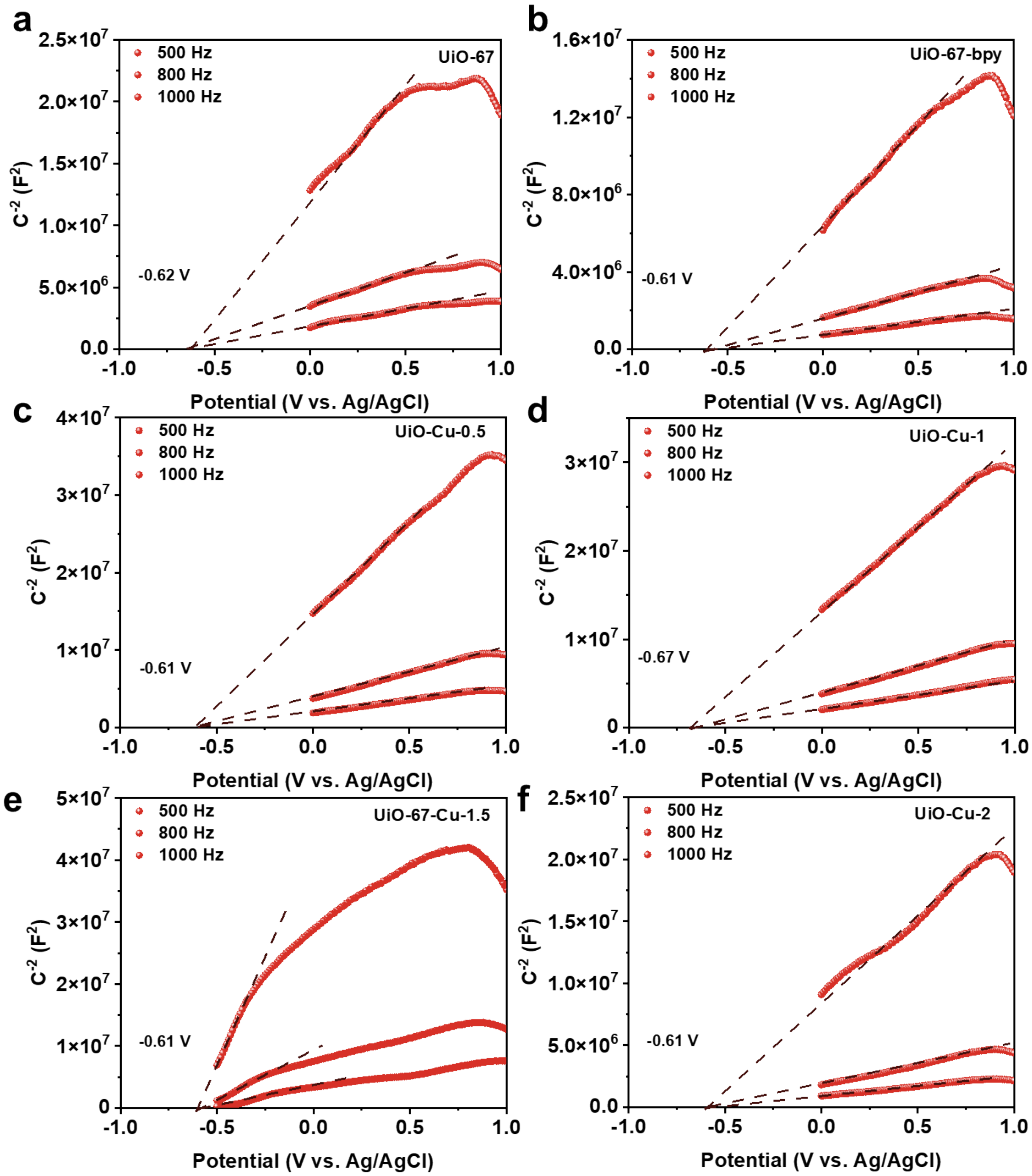


**Figure S27.** Mott-Schottky plots of **(a)** UiO-67, **(b)** UiO-67-bpy, **(c)** UiO-Cu-0.5, **(d)** UiO-Cu-1, **(e)** UiO-Cu-1.5, and **(f)** UiO-Cu-2 measured at different frequencies in 0.1 M $Na_2SO_4$ electrolyte (pH = 7).

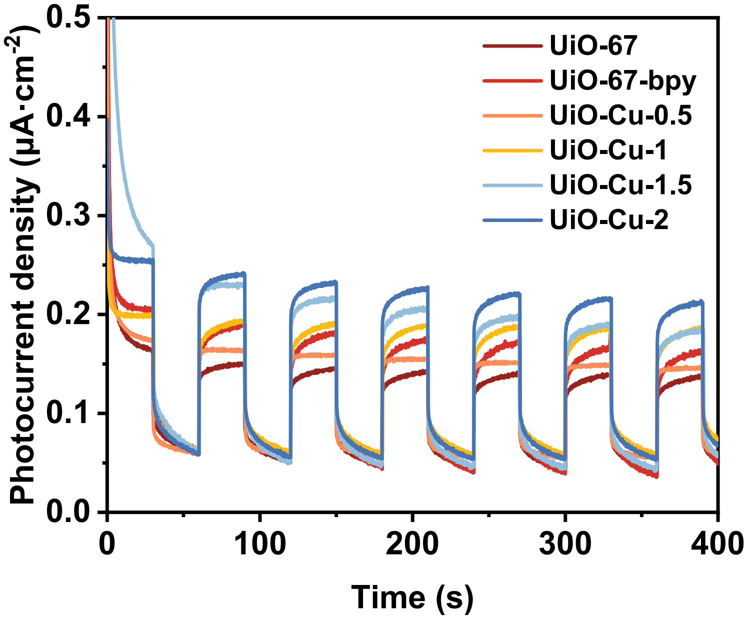


**Figure S28.** Transient photocurrent responses of UiO-67, UiO-67-bpy, and UiO-Cu-*x*.

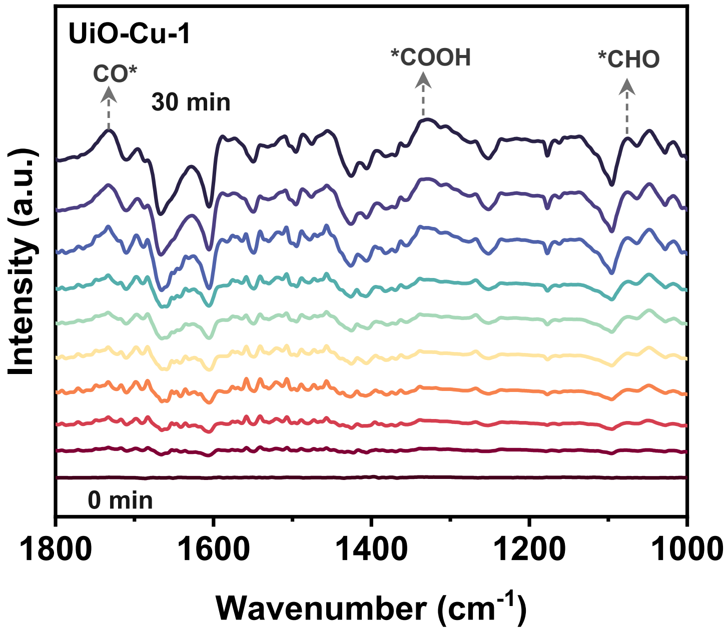


**Figure S29.** *In-situ* DRIFTS spectra for $CO_2$RR over UiO-Cu-1.

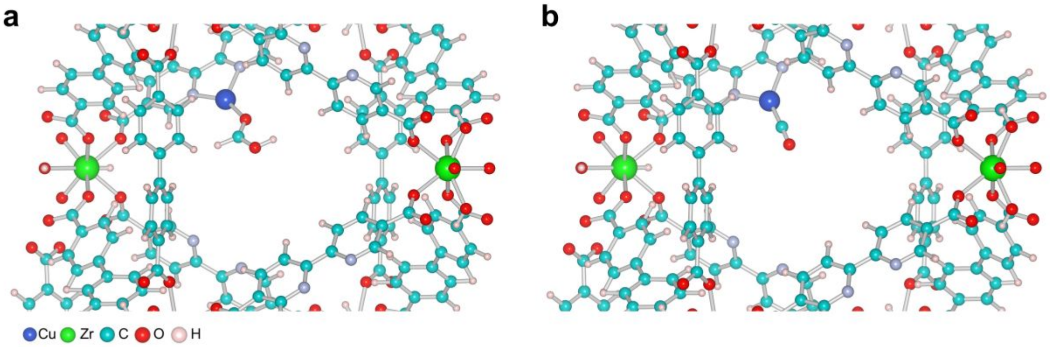


**Figure S30.** DFT-optimized structures of **(a)** *HCOOH and **(b)** *CO in the UiO-Cu-1.

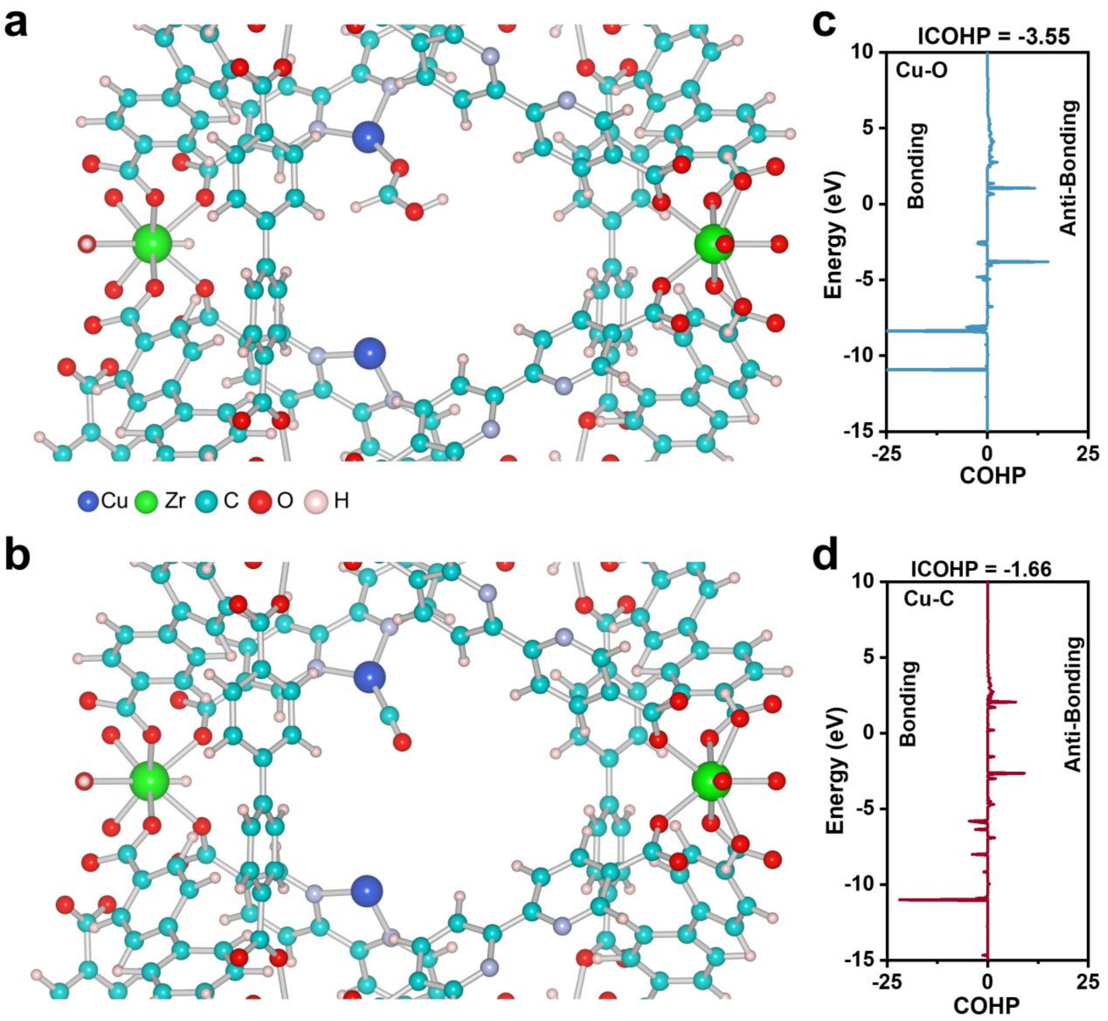


**Figure S31.** DFT-optimized structures of **(a)** *HCOOH-*□ and **(b)** *CO-*□ in the UiO-Cu-2. Corresponding COHP analysis of **(c)** Cu-O bond of the *HCOOH-*□, **(d)** Cu-C bond of the *CO-*□ in UiO-Cu-2.

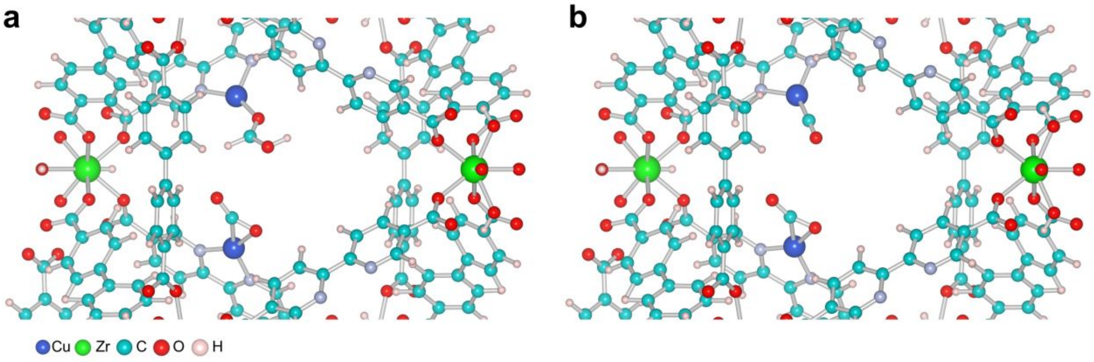


**Figure S32.** DFT-optimized structures of **(a)** *HCOOH-*$CO_2$ and **(b)** *CO-*$CO_2$ on the UiO-Cu-2.

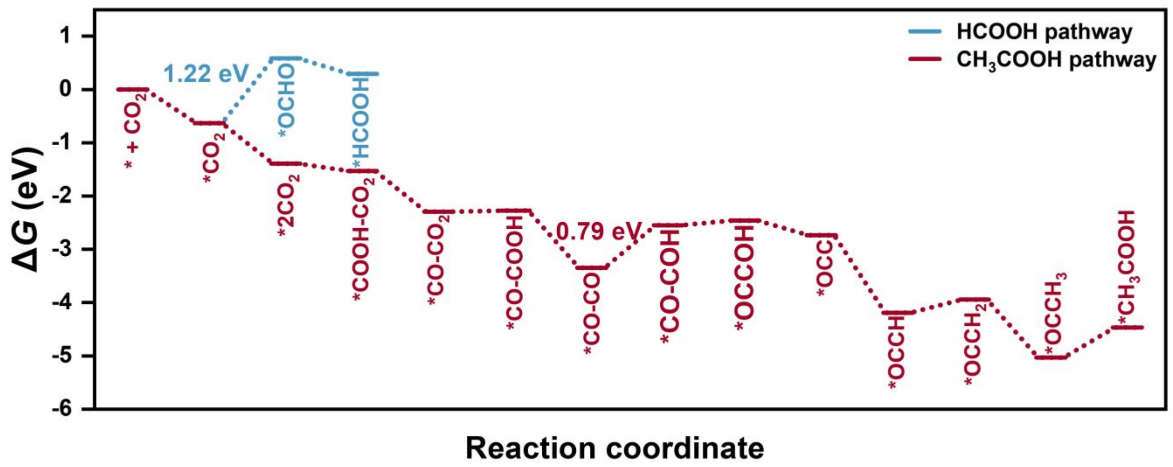


**Figure S33.** Gibbs free energy diagram for the $CO_2RR$ steps over UiO-Cu-2 with the RPBE functional.

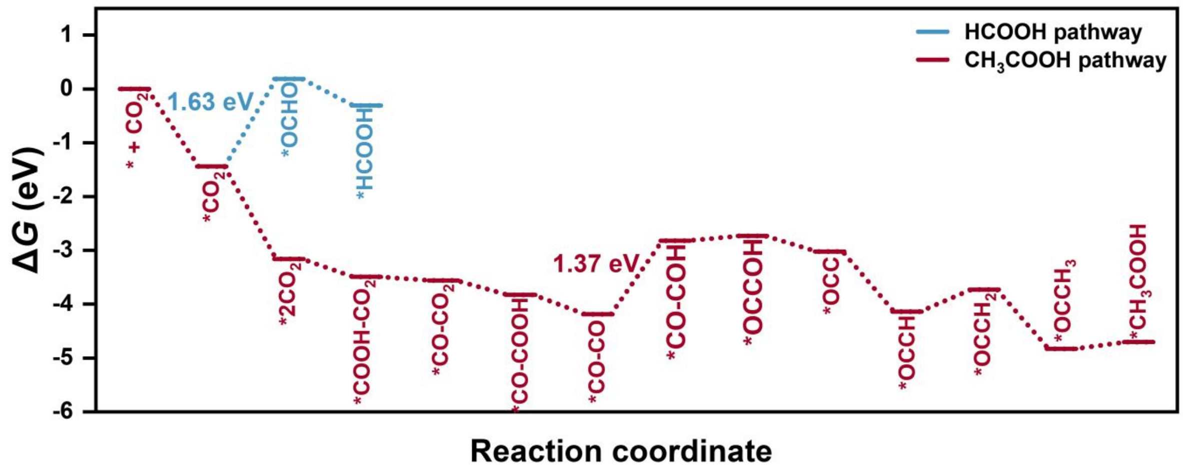


**Figure S34.** Gibbs free energy diagrams for the $CO_2RR$ steps over UiO-Cu-2 with implicit solvent effects.

In order to further confirm the rationality of the functional and model selection, we have added the Gibbs free energy diagrams calculated with the RPBE functional (**Figure S33**) method and implicit solvent effects (**Figure S34**), respectively. As shown in **Figure S33** and **Figure S34**, the energy changes calculated with the RPBE functional and implicit solvent effects are consistent with the previous conclusion, the model catalytic $CH_3COOH$ pathway has lower energy and is conducive

to the generation of $C_{2+}$ products, which proves the rationality of our DFT method selection. Furthermore, the results of the RPBE functional calculation significantly increased the energy differences (~0.43 eV) between two pathways, and the consistent trend has also been confirmed by previous reports (ACS Nano, 2023, 17, 24, 25667–25678).

**Table S1.** Crystallographic parameters derived from the PXRD measurements of UiO-67, UiO-67-bpy and UiO-Cu-0.5.

| | UiO-67 | UiO-67-bpy | UiO-Cu-0.5 |
|---|---|---|---|
| Radiation type (Å) | Cu Kα (1.5418) | Cu Kα (1.5418) | Cu Kα (1.5418) |
| 2θ range for refinement (°) | 4-60 | 4-60 | 4-60 |
| Space group | $Fm\bar{3}m$ | $Fm\bar{3}m$ | $Fm\bar{3}m$ |
| Crystal system | Cubic | Cubic | Cubic |
| a (Å) | 26.8524 | 26.8147 | 26.7943 |
| Cell volume (Å$^3$) | 19362.0 | 19280.7 | 19236.6 |
| Number of parameters | 26 | 28 | 26 |
| Number of *hkls* | 181 | 184 | 181 |
| Refinement method | Le Bail | Le Bail | Le Bail |
| $R_{wp}/R_{exp}/R_p$ (%) | 8.402/2.318/6.622 | 6.285/1.873/4.502 | 8.417/2.428/6.372 |
| *Gof* | 3.624 | 3.356 | 3.467 |

**Table S2.** Crystallographic parameters derived from the PXRD measurements of UiO-Cu-1, UiO-Cu-1.5 and UiO-Cu-2.

| | UiO-Cu-1 | UiO-Cu-1.5 | UiO-Cu-2 |
|---|---|---|---|
| Radiation type (Å) | Cu Kα (1.5418) | Cu Kα (1.5418) | Cu Kα (1.5418) |
| 2θ range for refinement (°) | 4-60 | 4-60 | 4-60 |
| Space group | $Fm\bar{3}m$ | $Fm\bar{3}m$ | $Fm\bar{3}m$ |
| Crystal system | Cubic | Cubic | Cubic |
| a (Å) | 26.8135 | 26.7984 | 26.8277 |
| Cell volume (Å) | 19278.0 | 19245.4 | 19308.6 |
| Number of parameters | 26 | 26 | 19 |
| Number of *hkls* | 182 | 182 | 184 |
| Refinement method | Le Bail | Le Bail | Le Bail |
| $R_{wp}/R_{exp}/R_p$ (%) | 7.471/2.001/5.716 | 7.762/1.999/5.856 | 8.417/2.428/6.372 |
| *Gof* | 3.732 | 3.883 | 3.467 |

**Table S3.** Elemental analysis of UiO-Cu-*x* by ICP-OES measurement.

| | Cu content (wt %) | Zr content (wt %) | Atomic ratio Cu : Zr |
|---|---|---|---|
| UiO-Cu-0.5 | 0.67 | 24.53 | 0.048:1 |
| UiO-Cu-1 | 1.38 | 22.49 | 0.088:1 |
| UiO-Cu-1.5 | 1.69 | 19.59 | 0.124:1 |
| UiO-Cu-2 | 2.00 | 19.38 | 0.149:1 |

**Table S4.** Results of BET analysis for all samples.

| Sample | BET surface area ($m^2/g$) | Adsorption average pore diameter (nm) | BJH adsorption average pore width (nm) |
|---|---|---|---|
| UiO-67 | 1017.15 | 2.5788 | 7.1641 |
| UiO-Cu-0.5 | 1293.38 | 2.4582 | 6.1877 |
| UiO-Cu-1 | 1209.05 | 2.4660 | 6.1921 |
| UiO-Cu-1.5 | 1188.01 | 2.5124 | 6.2039 |
| UiO-Cu-2 | 1436.71 | 2.4976 | 6.2281 |

**Table S5.** Curve fitting parameters for Cu K-edge EXAFS for UiO-Cu-1 and UiO-Cu-2.

| Sample | Path | N | R / Å | $\sigma^2$ / Å$^2$ | $\Delta E_0$ | R-factor | k range |
|---|---|---|---|---|---|---|---|
| UiO-Cu-1 | Cu-N/O | 4.0(6) | 1.94(1) | 0.004(2) | -4.7(21) | 0.015 | 3-13 |
| UiO-Cu-2 | Cu-N/O | 3.4(3) | 1.95(1) | 0.003(1) | -3.3(13) | 0.009 | 3-13 |

**Table S6.** Elemental analysis of UiO-Cu-2 by XPS measurement.

| | UiO-Cu-2 | | | | |
|---|---|---|---|---|---|
| Element | Cu 2p | O 1s | N 1s | C 1s | Zr 3d |
| Atomic % | 0.21 | 15.88 | 0.89 | 81.94 | 1.09 |

**Table S7.** Experimental EPR fitting parameters (g and A values) of UiO-Cu-1 and UiO-Cu-2, measured at 100 and 298 K.

| Samples | Temperature (K) | Species | $g_{\perp}$ | $g_{/\!/}$ | $A_{\perp}$ | $A_{/\!/}$ |
|---|---|---|---|---|---|---|
| UiO-Cu-1 | 100 | Cu-N(O); (**1**) | 2.080 | 2.245 | 95 | 550 |
| | | Cu-N(O); (**2**) | 2.084 | 2.200 | 85 | 400 |
| | 298 | Cu-N(O); (**1**) | 2.080 | 2.245 | 95 | 550 |
| | | Cu-N(O); (**2**) | 2.084 | 2.200 | 85 | 400 |
| UiO-Cu-2 | 100 | Cu-N(O); (**1**) | 2.080 | 2.245 | 95 | 550 |
| | | Cu-N(O); (**2**) | 2.084 | 2.200 | 85 | 400 |
| | | Cu···Cu; (**3**) | 2.069 | 2.235 | 30 | 550 |
| | 298 | Cu-N(O); (**1**) | 2.080 | 2.245 | 95 | 550 |
| | | Cu-N(O); (**2**) | 2.084 | 2.200 | 85 | 400 |
| | | Cu···Cu; (**3**) | 2.069 | 2.235 | 30 | 550 |

Note: **Species 1** and **2** for Cu–N(O)(1) and Cu–N(O)(2) produce EPR signals in both UiO-Cu-1 and UiO-Cu-2. **Species 3** for Cu···Cu interaction feature appears exclusively in the EPR spectrum of UiO-Cu-2.

**Table S8.** Quantitative peak parameters of samples from synchrotron PXRD.

| | UiO-67 | UiO-Cu-1 | UiO-Cu-2 |
|---|---|---|---|
| Peak asymmetry parameter | 0.0001 | 0.0001 | 0.0001 |
| LVol-Integral breadths | 113.9 | 157.8 | 104.4 |
| LVol-FWHM | 148.0 | 167.5 | 124.8 |
| Microstrain ($e_0$) * | 0.02279 | 0.02347 | 0.01096 |
| Strain (Gaussian) | 0.0911 | 0.0938 | 0.0438 |
| Strain (Lorentzian) | 0.0001 | 0.0001 | 0.0001 |

LVol - Volume weighted mean column heights.

$e_0$ - dislocations, vacancies, and other defects, calculated from FWHMs.

**Table S9.** Crystallographic data of the synchrotron PXRD measurements of UiO-67-bpy, UiO-Cu-1, and UiO-Cu-2.

| | UiO-67-bpy | UiO-Cu-1 | UiO-Cu-2 |
|---|---|---|---|
| X-ray energy (keV) | 16.99 | 16.99 | 16.99 |
| Beamline | BL02B2 | BL02B2 | BL02B2 |
| Detector | MYTHEN | MYTHEN | MYTHEN |
| Wavelength (Å) | 0.7296 | 0.7296 | 0.7296 |
| 2θ-zero point (°) | -0.00019 | -0.00019 | -0.00019 |
| Space group | $Fm\bar{3}m$ | $Fm\bar{3}m$ | $Fm\bar{3}m$ |
| Crystal system | Cubic | Cubic | Cubic |
| Cell volume (Å) | 19279.613 | 19283.350 | 19280.761 |
| a (Å) | 26.8143 | 26.8160 | 26.8148 |
| 2θ range for refinement (°) | 1.95-78.00 | 1.95-78.00 | 1.95-78.00 |
| Number of parameters | 49 | 42 | 43 |
| Number of *hkls* | 2617 | 2621 | 2617 |
| Refinement methods | Rietveld | Rietveld | Rietveld |
| $R_{wp}/R_{exp}/R_p$ (%) | 7.105/0.765/5.336 | 8.943/0.884/6.279 | 9.714/0.895/6.491 |

**Table S10.** Atomic parameters of UiO-67-bpy from Rietveld refinement.

| Atom | x | y | z | SOF | Beq (Å$^2$) | Wyckoff letter |
|---|---|---|---|---|---|---|
| Zr1 | -0.09195 | 0 | 0 | 1 | 1 | 24e |
| O1 | 0.12815 | 0 | 0.06626 | 1 | 1.5 | 96j |
| O2 | 0.06234 | 0.06234 | 0.06234 | 1 | 1.5 | 32f |
| C1 | 0.11680 | 0 | 0.11680 | 1 | 10 | 48h |
| C2 | 0.15662 | 0 | 0.15662 | 1 | 10 | 48h |
| C3 | 0.20633 | 0 | 0.14330 | 1 | 10 | 96j |
| C4 | 0.24272 | 0 | 0.17969 | 1 | 10 | 96j |
| C5 | 0.22940 | 0 | 0.22940 | 1 | 10 | 48h |
| C6 | 0.17969 | 0 | 0.24272 | 1 | 10 | 96j |
| C7 | 0.14330 | 0 | 0.20633 | 1 | 10 | 96j |

**Table S11.** Atomic parameters of UiO-Cu-1 from Rietveld refinement.

| Atom | x | y | z | SOF | Beq (Å$^2$) | Wyckoff letter |
|---|---|---|---|---|---|---|
| Zr1 | -0.08977 | 0 | 0 | 1 | 1 | 24e |
| O1 | 0.12877 | 0 | 0.06298 | 1 | 1.5 | 96j |
| O2 | 0.06234 | 0.06234 | 0.06234 | 1 | 1.5 | 32f |
| C1 | 0.11670 | 0 | 0.11670 | 1 | 10 | 48h |
| C2 | 0.15656 | 0 | 0.15652 | 1 | 10 | 48h |
| C3 | 0.20587 | 0 | 0.14329 | 1 | 10 | 96j |
| C4 | 0.24199 | 0 | 0.17942 | 1 | 10 | 96j |
| C5 | 0.22877 | 0 | 0.22877 | 1 | 10 | 48h |
| C6 | 0.17942 | 0 | 0.24199 | 1 | 10 | 96j |
| C7 | 0.14329 | 0 | 0.20587 | 1 | 10 | 96j |
| Cu | 0.31647 | 0 | 0.17552 | 0.07 | 10 | 96j |

**Table S12.** Atomic parameters of UiO-Cu-2 from Rietveld refinement.

| Atom | x | y | z | SOF | Beq (Å$^2$) | Wyckoff letter |
|---|---|---|---|---|---|---|
| Zr1 | -0.09007 | 0 | 0 | 1 | 1 | 24e |
| O1 | 0.12729 | 0 | 0.06948 | 1 | 1.5 | 96j |
| O2 | 0.06234 | 0.06234 | 0.06234 | 1 | 1.5 | 32f |
| C1 | 0.11670 | 0 | 0.11670 | 1 | 10 | 48h |
| C2 | 0.15652 | 0 | 0.15652 | 1 | 10 | 48h |
| C3 | 0.20587 | 0 | 0.14330 | 1 | 10 | 96j |
| C4 | 0.24200 | 0 | 0.17942 | 1 | 10 | 96j |
| C5 | 0.22877 | 0 | 0.22877 | 1 | 10 | 48h |
| C6 | 0.17942 | 0 | 0.24200 | 1 | 10 | 96j |
| C7 | 0.14330 | 0 | 0.20587 | 1 | 10 | 96j |
| Cu | 0.31648 | 0 | 0.17552 | 0.15 | 10 | 96j |

**Table S13.** TON value for the total product of catalysts in different Cu concentrations.

| Catalyst | $TON_{total}$* | $TON_{acetic\ acid}$ |
|---|---|---|
| UiO-Cu-0.5 | 2.25 | 0 |
| UiO-Cu-1 | 1.53 | 0 |
| UiO-Cu-1.5 | 0.44 | 0.20 |
| UiO-Cu-2 | 0.96 | 0.77 |

* TON was calculated using 30 mg of catalyst with 5 hours of illumination.

**Table S14.** Photocatalytic performance for the direct conversion of $CO_2$ to acetic acid.

| Photocatalyst | Reaction parameters | Other products | The formation rate of $CH_3COOH$ | Ref |
|---|---|---|---|---|
| NiCo-$TiO_2$ | 300 W Xe lamp, 0.1 m $Na_2SO_3$ and 0.2 m CsOH | $CH_3OH$, $CH_4$, CO | 22.6 $\mu molg^{-1}h^{-1}$ (71 %) | 1 |
| 3%Cu/$WO_3$ | 300 W Xe lamp, deionized water | CO, $CH_4$, HCOOH, $H_2O_2$ | 2.87 $\mu molg^{-1}h^{-1}$ (67 %) | 2 |
| $Cu_2Pt_2/WO_3$ | 300 W Xe lamp, 20 mL water with 15 mmol $L^{-1}$ HCl | HCOOH, CO, $CH_4$, $CH_3OH$, $H_2O_2$ | 19.41$\mu molg^{-1}h^{-1}$ (88.1 %) | 3 |
| ILs-TNTs | 500 W Xe lamp, deionized water | HCOOH, $H_2$ | 12.4 $\mu molg^{-1}h^{-1}$ (75 $\%_{\text{carbon product}}$) | 4 |
| 1%AgCl@g-$C_3N_4$ | 11 W compact fluorescent lamp, TEA (0.1 M) medium | $CH_4$, HCOOH | 1.2 $\mu molg^{-1}h^{-1}$ (< 10 %) | 5 |
| 3%$Nb_2O_5$/TNTs | 300 W Xe lamp, deionized water | HCOOH, $CH_3OH$, $CH_3CH_2OH$ | 28.4 $\mu molg^{-1}h^{-1}$ (69 %) | 6 |
| *d*-UiO-66/$MoS_2$ | 300 W Xe lamp with a UV cut-off filter ($\lambda \geq$ 400 nm), deionized water, 99.99% $CO_2$ | $O_2$, $CH_3CH_2OH$ | 39.0 $\mu molg^{-1}h^{-1}$ (93 $\%_{\text{carbon product}}$) | 7 |
| Pd-$Co_3O_4$ | Xe lamp, with a standard AM 1.5G filter (100 $mW/cm^2$), film | CO | 13.8 $\mu molg^{-1}h^{-1}$ (78 %) | 8 |
| $In_2S_3$-C/$Fe_3C$ | 300 W Xe lamp with AM 1.5 G filter, 0.1 M $KHCO_3$ solution | HCOOH, $CH_3CH_2OH$ | 11.33 $\mu molg^{-1}h^{-1}$ (89 %) | 9 |
| UiO-Cu-2 | 300 W Xe lamp (53.5 $mW/cm^2$), $H_2O$/TEOA (500:1, v/v) | CO, $CH_4$ | 48.6 $\mu molg^{-1}h^{-1}$ (89 %) | This work |

**Table S15.** Results of the hot filtration test: Comparison of catalytic activity before and after catalyst removal.

| Condition | After 5 h irradiation (with catalyst) | After subsequent 24 h in dark (catalyst filtered) | After re-irradiation for 5h (catalyst filtered) |
|---|---|---|---|
| $^1$H NMR area | 22883.03 | 20462.39 | 21696.12 |

**Table S16.** Surface area analysis for post-mortem UiO-Cu-2.

| Sample | BET surface area ($m^2$/g) | Adsorption average pore diameter (nm) | BJH adsorption average pore width (nm) |
|---|---|---|---|
| UiO-Cu-2 | 139.91 | 7.2678 | 8.9048 |

**Table S17.** Elemental analysis of UiO-Cu-2 after $CO_2$RR from ICP-OES measurement.

| Sample | Test element | Leaching concentration (ppm) | Leaching content (mg for Cu) | Leaching percentage (wt%) |
|---|---|---|---|---|
| UiO-Cu-2 | Cu | 0.5453 | 0.0273 | 3.13 |

**Table S18.** Curve fitting parameters for Cu K-edge EXAFS for post-catalytic UiO-Cu-2.

| Sample | Path | N | R / Å | $\sigma^2$ / Å$^2$ | $\Delta E_0$ | R-factor | k range |
|---|---|---|---|---|---|---|---|

| | | | | | | | |
|---|---|---|---|---|---|---|---|
| After | Cu-N/O | 4.1(8) | 1.95(2) | 0.006(3) | -3.0(26) | 0.019 | 3-10 |

**Table S19.** Band positions of the as-prepared samples vs. NHE.

| Sample | Bandgap (eV) | Valence band (eV) | Conduction band (eV) |
|---|---|---|---|
| UiO-67 | 3.68 | 3.37 | -0.31 |
| UiO-67-bpy | 3.72 | 3.42 | -0.30 |
| UiO-Cu-0.5 | 3.56 | 3.26 | -0.30 |
| UiO-Cu-1 | 3.51 | 3.15 | -0.36 |
| UiO-Cu-1.5 | 3.50 | 3.20 | -0.30 |
| UiO-Cu-2 | 3.47 | 3.16 | -0.31 |

**Table S20.** Lifetime components in time-resolved PL (TRPL) decay fitted by double exponential components.

| Sample | $\tau_1$ (ns) | $A_1$ (%) | $\tau_2$ (ns) | $A_2$ (%) | $T_{eff}$ |
|---|---|---|---|---|---|
| UiO-67 | 0.965 | 73.51 | 4.145 | 26.49 | 1.808 |
| UiO-67-bpy | 0.767 | 85.01 | 4.660 | 14.99 | 1.350 |
| UiO-Cu-0.5 | 0.691 | 88.04 | 3.268 | 11.96 | 1.000 |
| UiO-Cu-1 | 0.689 | 85.99 | 3.404 | 14.01 | 1.069 |
| UiO-Cu-1.5 | 0.679 | 87.99 | 3.396 | 12.01 | 1.006 |
| UiO-Cu-2 | 0.633 | 85.64 | 3.369 | 14.36 | 1.026 |

All decay profiles were fitted using a bi-exponential function $y = A_1 \exp(-\frac{t}{\tau_1}) + A_2 \exp(-\frac{t}{\tau_2})$, yielding two distinct lifetime components ($\tau_1$ and $\tau_2$). The effective lifetime is ($\tau_{eff} = (A_1\tau_1 + A_2\tau_2)/(A_1 + A_2)$) is extracted from the TRPL spectra by fitting with a biexponential function (**Table S20**). TRPL lifetime reflects the average survival time of photogenerated electron-hole pairs. UiO-67, UiO-67-bpy, and UiO-Cu-*x* (*x*= 0.5, 1, 1.5 and 2) have a $\tau_{eff}$ of 1.808, 1.350, 1.000, 1.069, 1.006 and 1.026 ns, respectively. This trend aligns with enhanced charge separation and migration to catalytic sites, rather than detrimental recombination within the bulk structure. The shortened TRPL lifetimes, coupled with reduced steady-state PL intensity in UiO-Cu-x catalysts, strongly correlate with the introduction of copper species. These dual observations with shorter TRPL lifetimes and lower PL intensity suggest that copper incorporation promotes efficient extraction of carriers for surface redox reactions, suppressing self-recombination. Further analysis of the biexponential fitting reveals two recombination pathways: the faster decay component ($\tau_1$) relates to non-radiative recombination mediated by trap states, while the slower decay component ($\tau_2$) reflects intrinsic radiative recombination of free carriers. Pristine UiO-67 exhibits the longest $\tau_1$ (0.965 ns), attributed to ligand-to-cluster charge transfer (LCCT) processes. Substitution with

bipyridine ligands in UiO-67-bpy reduces $\tau_1$ to 0.767 ns, likely due to nitrogen-induced charge delocalization. Remarkably, UiO-Cu-*x* catalysts display the shortest $\tau_1$ values (0.633-0.691 ns) and the highest contribution of the fast-decay component ($A_1$), consistent with ligand-to-metal charge transfer (LMCT) dynamics. This LMCT behavior, facilitated by the rigid metal-organic framework, promotes rapid exciton quenching through directional charge migration. The controlled integration of copper into the UiO-67 framework likely induces localized charge asymmetry, driving spatial separation of electrons and holes toward active catalytic sites. This mechanistic framework explains the superior photocatalytic $CO_2$ reduction performance of UiO-Cu-*x*, where optimized carrier utilization outweighs the reduced bulk recombination lifetime.

**References**

(1) Jia, G.; Sun, M.; Wang, Y.; Shi, Y.; Zhang, L.; Cui, X.; Huang, B.; Yu, J. C. Asymmetric Coupled Dual-Atom Sites for Selective Photoreduction of Carbon Dioxide to Acetic Acid. *Advanced Functional Materials* **2022**, *32* (41), 2206817. DOI: 10.1002/adfm.202206817.

(2) Zeng, D.; Wang, H.; Zhu, X.; Cao, H.; Zhou, Y.; Wang, W.; Zhang, L.; Wang, W. Single-atom copper modified hexagonal tungsten oxide for efficient photocatalytic $CO_2$ reduction to acetic acid. *Chemical Engineering Journal* **2023**, *451*, 138801. DOI: 10.1016/j.cej.2022.138801.

(3) Zeng, D.; Wang, H.; Zhu, X.; Cao, H.; Wang, W.; Zhang, Y.; Wang, J.; Zhang, L.; Wang, W. Photocatalytic conversion of $CO_2$ to acetic acid by CuPt/$WO_3$: Chloride enhanced C-C coupling mechanism. *Applied Catalysis B: Environmental* **2023**, *323*, 122177. DOI: 10.1016/j.apcatb.2022.122177.

(4) Lv, R.; Zhang, M.; Huang, H.; Yuan, S.; Liu, L.; Li, K.; Yuan, J.; Hu, H. Self-doped $TiO_2$ nanotubes with surface modification by ionic liquids for enhanced photoreduction of $CO_2$ to acetic acid. *Applied Surface Science* **2023**, *621*, 156897. DOI: 10.1016/j.apsusc.2023.156897.

(5) Murugesan, P.; Narayanan, S.; Manickam, M.; Murugesan, P. K.; Subbiah, R. A direct Z-scheme plasmonic AgCl@g-$C_3N_4$ heterojunction photocatalyst with superior visible light $CO_2$ reduction in aqueous medium. *Applied Surface Science* **2018**, *450*, 516-526. DOI: 10.1016/j.apsusc.2018.04.111.

(6) Lv, R.; Liu, K.; Hu, H.; Fan, M.; Li, K.; Zhang, M.; Huang, H. Boosting $CO_2$ photoreduction to acetic acid via the van der waals heterostructures of monolayer $Nb_2O_5$ modified $TiO_2$ nanotubes. *Separation and Purification Technology* **2025**, *359*, 130835. DOI: 10.1016/j.seppur.2024.130835.

(7) Yu, F.; Jing, X.; Wang, Y.; Sun, M.; Duan, C. Hierarchically Porous Metal-Organic Framework/$MoS_2$ Interface for Selective Photocatalytic Conversion of $CO_2$ with $H_2O$ into $CH_3$

COOH. *Angewandte Chemie International Edition* **2021**, *60* (47), 24849-24853. DOI: 10.1002/anie.202108892.

(8) Ding, J.; Du, P.; Zhu, J.; Hu, Q.; He, D.; Wu, Y.; Liu, W.; Zhu, S.; Yan, W.; Hu, J.; et al. Light-Driven C-C Coupling for Targeted Synthesis of $CH_3COOH$ with Nearly 100% Selectivity from $CO_2$. *Angewandte Chemie International Edition* **2024**, *63* (13), e202400828. DOI: 10.1002/anie.202400828 .

(9) Liao, C.; Jing, W.; Wang, F.; Liu, Y. 3D $In_2S_3/C/Fe_3C$ nanofibers for Z-scheme photocatalytic $CO_2$ conversion to acetate. *Materials Today Catalysis* **2023**, *3*, 100030. DOI: 10.1016/j.mtcata.2023.100030.
